# Exome-wide association analysis reveals novel coding sequence variants associated with lipid traits in Chinese

**DOI:** 10.1038/ncomms10206

**Published:** 2015-12-22

**Authors:** Clara S. Tang, He Zhang, Chloe Y. Y. Cheung, Ming Xu, Jenny C. Y. Ho, Wei Zhou, Stacey S. Cherny, Yan Zhang, Oddgeir Holmen, Ka-Wing Au, Haiyi Yu, Lin Xu, Jia Jia, Robert M. Porsch, Lijie Sun, Weixian Xu, Huiping Zheng, Lai-Yung Wong, Yiming Mu, Jingtao Dou, Carol H. Y. Fong, Shuyu Wang, Xueyu Hong, Liguang Dong, Yanhua Liao, Jiansong Wang, Levina S. M. Lam, Xi Su, Hua Yan, Min-Lee Yang, Jin Chen, Chung-Wah Siu, Gaoqiang Xie, Yu-Cho Woo, Yangfeng Wu, Kathryn C. B. Tan, Kristian Hveem, Bernard M. Y. Cheung, Sebastian Zöllner, Aimin Xu, Y Eugene Chen, Chao Qiang Jiang, Youyi Zhang, Tai-Hing Lam, Santhi K. Ganesh, Yong Huo, Pak C. Sham, Karen S. L. Lam, Cristen J. Willer, Hung-Fat Tse, Wei Gao

**Affiliations:** 1 Department of Psychiatry, the University of Hong Kong, Hong Kong, China; 2 Department of Internal Medicine, Division of Cardiovascular Medicine, University of Michigan, Ann Arbor, Michigan 48109, USA; 3 Department of Medicine, the University of Hong Kong, Hong Kong, China; 4 Department of Cardiology, Institute of Vascular Medicine, Peking University Third Hospital, Key Laboratory of Molecular Cardiovascular Sciences, Ministry of Education, Beijing 100191, China; 5 Department of Computational Medicine and Bioinformatics, University of Michigan, Ann Arbor, Michigan 48109, USA; 6 Centre for Genomic Sciences, Li Ka Shing Faculty of Medicine, The University of Hong Kong, Hong Kong, China; 7 State Key Laboratory of Brain and Cognitive Sciences, The University of Hong Kong, Hong Kong, China; 8 Department of Cardiology, Peking University First Hospital, Beijing 100034, China; 9 Department of Public Health and General Practice, HUNT Research Centre, Norwegian University of Science and Technology, 7600 Levanger, Norway; 10 St Olav Hospital, Trondheim University Hospital, 7030 Trondheim, Norway; 11 School of Public Health, the University of Hong Kong, Hong Kong, China; 12 Department of Endocrinology, Chinese People's Liberation Army General Hospital, Beijing 100853, China; 13 Beijing Hypertension League Institute, Beijing 100039, China; 14 Peking University Shougang Hospital, Beijing, China; 15 Department of Cardiology, Wuhan Asia Heart Hospital, China; 16 Research Centre of Heart, Brain, Hormone and Healthy Aging, Li Ka Shing Faculty of Medicine, The University of Hong Kong, Hong Kong, China; 17 Peking University Clinical Research Institute, Beijing, China; 18 Peking University Clinical Research Institute, Department of Epidemiology and Biostatistics, Peking University School of Public Health, Beijing, China; 19 State Key Laboratory of Pharmaceutical Biotechnology, The University of Hong Kong, Hong Kong, China; 20 Department of Biostatistics, Center for Statistical Genetics, University of Michigan School of Public Health, Ann Arbor, Michigan 48109, USA; 21 Department of Pharmacology & Pharmacy, The University of Hong Kong, Hong Kong, China; 22 Guangzhou No.12 Hospital, Guangzhou 510620, China; 23 Institute of Vascular Medicine, Peking University Third Hospital, Beijing Key Laboratory of Cardiovascular Receptors Research, Beijing 100191, China; 24 Department of Human Genetics, University of Michigan, Ann Arbor, Michigan 48109, USA; 25 Hong Kong-Guangdong Joint Laboratory on Stem Cell and Regenerative Medicine, the University of Hong Kong, Hong Kong, China; 26 Department of Cardiology, Peking University Third Hospital, Key Laboratory of Cardiovascular Molecular Biology and Regulatory Peptides, Ministry of Health, Beijing 100191, China

## Abstract

Blood lipids are important risk factors for coronary artery disease (CAD). Here we perform an exome-wide association study by genotyping 12,685 Chinese, using a custom Illumina HumanExome BeadChip, to identify additional loci influencing lipid levels. Single-variant association analysis on 65,671 single nucleotide polymorphisms reveals 19 loci associated with lipids at exome-wide significance (*P*<2.69 × 10^−7^), including three Asian-specific coding variants in known genes (*CETP* p.Asp459Gly, *PCSK9* p.Arg93Cys and *LDLR* p.Arg257Trp). Furthermore, missense variants at two novel loci—*PNPLA3* p.Ile148Met and *PKD1L3* p.Thr429Ser*—*also influence levels of triglycerides and low-density lipoprotein cholesterol, respectively. Another novel gene, *TEAD2*, is found to be associated with high-density lipoprotein cholesterol through gene-based association analysis. Most of these newly identified coding variants show suggestive association (*P*<0.05) with CAD. These findings demonstrate that exome-wide genotyping on samples of non-European ancestry can identify additional population-specific possible causal variants, shedding light on novel lipid biology and CAD.

## Abstract (web summary)

An important risk factor for coronary artery disease is the level of blood lipids. Here the authors conduct an exome-wide association study in Chinese cohorts and identify three novel loci associated with lipid levels as well as three Asian-specific variants in known loci.

## Graphical abstract

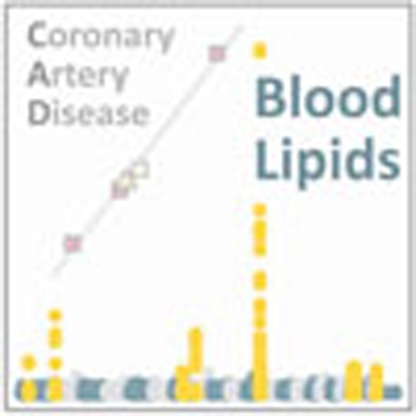

Dyslipidemia, often defined as elevated levels of total cholesterol (TC), triglyceride (TG) or low-density lipoprotein cholesterol (LDL-C) or decreased level of high-density lipoprotein cholesterol (HDL-C), is an important risk factor for coronary artery disease (CAD)^1^ which is a major cause of mortality and morbidity worldwide^2^. The incidence of CAD and the prevalence of its major risk factors, such as dyslipidemia, vary widely according to geographical location and ethnicity^3^. Such differences could be due to environmental factors, like smoking and diet, or underlying population genetic differences. Mapping of the genetic loci affecting blood lipid levels may shed light on disease pathogenesis and provide pharmaceutical targets for prevention and treatment of CAD.

Recently, a multi-ethnic meta-analysis of genome-wide association studies (GWAS) on >188K individuals, the majority being of European ancestry, discovered a total of 157 loci associated with blood lipid levels that together accounted for about 20–30% of the total genetic contribution^4^. Most of these variants are common, non-coding, and with modest impact on lipid levels, although rare coding variants with larger effects have been identified in a number of genes, including *PCSK9* (refs 5, 6), *LDLR*^5^,^7^, *APOC3* (refs 8, 9) and *APOB*^4^,^6^,^10^. By exploiting the differences in linkage disequilibrium (LD) of the non-European populations (2–9K), the study further narrowed down association signals at five loci, corroborating findings of fine-mapping analyses on Europeans with a much larger sample size.

The vast majority of discovery efforts have been made in samples of European ancestry^4^,^6^,^11^, which contain only a subset of human genetic variation^12^. The degree of overlap between the variants and genes that contribute to lipid heritability in different populations is largely unexplored. During evolutionary history, genetic drift and evolutionary selection have led to different genetic architectures for populations in different geographic areas^13^. For example, variants in *APOC3* demonstrate higher frequencies in isolated populations with different ancestry, notably Greeks^14^ and Amish^15^. Furthermore, loss-of-function mutations in *PCSK9* are more prevalent in individuals with African ancestry^5^,^6^. Inter-population differences in allele frequencies may therefore affect the detectability of risk variants^16^. This highlights the importance of association analyses on non-European populations to identify novel loci influencing lipid levels.

To systematically evaluate the contribution of coding variants to lipid levels in an Asian population, we perform an exome-wide association analysis using a custom Asian Exomechip^17^ on 12,685 individuals selected from 2 Chinese cohorts, the University of Hong Kong Theme-based Research Scheme (HKU-TRS, *N*=5,233) and Peking University Health Science Center and the University of Michigan Medical School study of Myocardial Infarction (PUUMA-MI, *N*=7,452). First, we address whether known lipid variants identified in Europeans are associated with blood lipid levels in East Asian samples. Then we compare the effect size and power in detecting known variants between HKU-TRS and a Norwegian study of similar sample size (Nord-Trøndelag Health Study (HUNT) study, *N*=5,643). Next, we seek to identify coding variants in known loci that may point to the functional lipid genes and explore other protein-altering variants for novel lipid-associated genes in this Asian population. We discover a total of three Asian-specific associations involving rare nonsynonymous changes and identify three novel lipid-associated loci not previously reported. Our study demonstrates the importance of ancestry-specific association analysis to discover population-specific associations of rare variants and, most importantly, to provide new insights into novel biological pathways for lipid metabolism.

## Results

Using an exome array augmented with custom markers, we successfully genotyped 145,276 polymorphic single nucleotide polymorphisms (SNPs) in 12,685 Chinese individuals (Supplementary Table 1). Single-variant and gene-based association analyses were carried out with respect to the four blood lipid levels (LDL-C, HDL-C, TC and TG). Single-variant score test statistics were presented for the following analyses, unless stated otherwise. Specifically, we assessed association of 65,671 variants that were polymorphic in both Chinese cohorts and had at least 20 minor alleles (MAC⩾20). Of these variants, 58% altered protein composition and 26% were Asian-specific variants with minor allele frequency (MAF) <5% (Supplementary Table 2).

### Power and effect size comparison with HUNT-MI

We initially examined the known European-identified GWAS variants^4^,^11^ in our combined data set (*N*=12,685). Of the 157 index SNPs, 123 variants, including 4 with MAC<20, were successfully genotyped. We then tested 119 variants for association with their primary lipid traits. The analysis identified 8 variants reaching genome-wide significance (*P*<5 × 10^−8^) and 52 reaching nominal significance (*P*<0.05). The large proportions of associated variants were substantially more than expected under the null hypothesis of no association (binomial test *P*=3.1 × 10^−47^ and *P*=1.5 × 10^−35^, respectively; Supplementary Table 3). Approximately 83% of the variants (*N*=99) had direction of effect concordant to those reported (Supplementary Fig. 1).

To explore if differences in LD may affect power for detecting association of the known GWAS SNPs in Asian samples, we compared the number of significant variants discovered in a Norwegian cohort—HUNT-MI (*N*=5,643)^18^—against one of our Chinese cohorts—HKU-TRS (*N*=5,233)—of similar sample size. Compared with HUNT-MI, fewer significant associations were detected in HKU-TRS (4 versus 7 for genome-wide significant variants and 32 versus 45 for variants with binomial test *P*<0.05; Supplementary Table 4), suggesting a slight decrease in power to detect known European-identified variants in Asian samples. Such differences might be due to LD differences between the index SNP and the true causal variant. This hypothesis was supported by a weaker correlation with the reported effect sizes for Chinese at three of the four lipid traits (HKU-TRS: LDL-C *r*^2^=0.49, HDL-C *r*^2^=0.61, TC *r*^2^=0.13 and TG *r*^2^=0.62) than Norwegian (HUNT-MI: LDL-C *r*^2^=0.46, HDL-C *r*^2^=0.75, TC *r*^2^=0.47 and TG *r*^2^=0.84).

### Refining association signals at known loci

Differences in LD patterns, on the one hand, may affect the detectability of variants tagging the casual SNP; on the other hand, it may help pinpoint the causal variant. We next investigated if variants other than the index SNPs could account for the association of the previously established loci, with the caveat that the majority of the variants we tested are coding. Including the eight loci with significant GWAS SNPs, a total of 16 known loci were associated with at least 1 lipid trait at exome-wide significance (*P*<2.69 × 10^−7^) (Supplementary Figs 2–6; Supplementary Tables 5–8). The association was evaluated after conditioning on nearby index SNP(s), and *vice versa*, revealing that only signals of four loci (*ABCA1* for HDL-C, *DOCK7-ANGPTL3*, *GCKR* and *MLXIPL* for TG) could be completely explained by the GWAS index SNPs (Supplementary Data 1). This list could be further extended to include *LIPC* if variants reported to show independent associations were considered as well^18^.

One of our non-index lead SNPs, rs13702 at the 3′ untranslated region of *LPL*, exhibited stronger association with HDL-C (*P*=1.3 × 10^−18^) than the GWAS SNP (rs12678919, *r*^2^=0.47, *P*=5.7 × 10^−15^). Association of the GWAS SNP was largely attenuated (conditional *P*=4.0 × 10^−3^) once accounted for the effect of rs13702, whereas rs13702 still showed near exome-wide significant association after conditional analysis (conditional *P*=7.2 × 10^−7^). This suggested that the *LPL* index SNP might act as a proxy for rs13702. Refinement of association signals by population differences in LD could be further demonstrated by our lead SNPs at *CELSR2*-*SORT1* for LDL-C (rs12740374, *P*=4.9 × 10^−18^) and *APOA5* for TG (rs651821, *P*=5.0 × 10^−102^), where both SNPs were highlighted as the new lead SNPs in previous fine-mapping analyses involving African and East Asian populations, respectively^4^. Likewise, our lead SNP encoding TM6SF2 p.Glu167Lys (rs58542926, *P*=1.5 × 10^−8^) was recently shown to be a causal variant altering TC and TG levels^18^,^19^.

### New lead SNPs at APOB and DOCK6

Besides refining known signals, some of the new lead SNPs appeared largely independent of the index SNPs (*r*^2^<0.03), representing novel associations not previously reported (Table 1). At *APOB*, the new lead SNP corresponded to a novel missense variant (rs13306194, encoding p.Arg532Trp) associated with decreasing LDL-C (*β*=−0.134 s.d., *P*=1.2 × 10^−12^) and TC levels (*β*=−0.129, *P*=6.0 × 10^−12^), whereas the GWAS index SNP was only weakly associated with both lipid traits (*P*>4 × 10^−3^). Interestingly, although *APOB* p.Arg532Trp is relatively common among Asians (MAF=0.13), it has been found to be extremely rare in 1000 Genomes European (MAF=0.003) and African samples (MAF=0.004), as well as in HUNT-MI Norwegians (MAF=7 × 10^−4^). Similarly, a synonymous *DOCK6* variant (rs737337, encoding p.Thr712Thr, MAF=0.27), known to be associated with HDL-C, was associated with TC level (*P*=7.5 × 10^−10^) in our study and displayed a large difference in allele frequency across populations (MAF=0.07 in Europeans). Only marginal association has hitherto been reported with TC.

### Complex association at APOE and APOA5

For two of the previously implicated loci, Apolipoprotein E (*APOE*) and *APOA5*, we observed not only one but multiple independent association signals. In some cases, a different lead SNP was observed for different lipid traits. *APOE*, located in the apolipoprotein family gene cluster (*APOE–APOC1*–*APOC2*–*APOC4*), showed significant association with all lipid traits under study. Stepwise conditional analysis revealed two exome-wide significant independent associations with HDL-C, including an intronic lead SNP (rs769449, MAF=0.08, *P*=3.8 × 10^−10^) and a known independent SNP encoding APOC4 p.Leu96Arg (rs5167, MAF=0.45, *P*=1.6 × 10^−7^) (Supplementary Fig. 7). The intronic lead SNP is in moderately strong LD with the index SNP (rs4420638, *r*^2^=0.68, *P*=0.002), whereas *APOC4* p.Leu96Arg is not linked to either SNP in the joint regression model (all *r*^2^<6 × 10^−4^). For other lipid traits, a different lead SNP (rs445925), also a known independent SNP associated with LDL-C, was observed (*P*_LDL-C_=1.9 × 10^−64^, *P*_TC_=4.1 × 10^−26^, *P*_TG_=7.1 × 10^−9^). For all except TG, the associations were significantly independent, though the effects were largely reduced after conditioning on the reported SNPs.

For the *APOA5* locus, as mentioned previously, the strongest association signal for TG and HDL-C mapped to the GWAS fine-mapped SNP (rs651821, or a proxy SNP for HDL-C (rs662799, *r*^2^=0.99))^4^ (Supplementary Figs 8 and 9). The secondary association signal for HDL-C was originally masked (rs10466588; unconditional *P*=2.9 × 10^−4^ to conditional *P*=5.8 × 10^−11^) by the strong effect of the index SNPs, but was later discovered through stepwise conditional analysis. By further conditioning on rs10466588, we identified the second independent signal in the region, which encodes APOA5 p.Gly185Cys (rs2075291; unconditional *β*=−0.3, *P*=1.2 × 10^−29^ to conditional *β*=−0.38, *P*=1.9 × 10^−7^). This missense change is near Asian-specific with MAF of ∼6%, but is present in low frequency in Africans (MAF=0.2%) and not observed in Europeans.

### Asian-specific association at CETP, LDLR and PCSK9

In addition, we also identified significant association of three Asian-specific missense variants defined as being polymorphic in Asian but monomorphic in other populations (Table 1). This included a low-frequency missense *CETP* SNP independently associated with HDL-C (rs2303790, p.Asp459Gly, MAF=0.027; *β*=0.44, *P*=3.2 × 10^−29^). For LDL-C, associations with strong effect were detected for two probably damaging missense changes—a rare *PCSK9* variant encoding p.Arg93Cys (rs151193009, MAF=0.014; *P*=7.9 × 10^−32^) and a *LDLR* mutation encoding p.Arg257Trp (rs200990725, MAF=0.001; *P*=3.0 × 10^−8^). Both variants appeared to be independent of previously reported association signals. The *PCSK9* p. Arg46Leu missense variant (rs11591147) was near monomorphic in Chinese and the GWAS index SNP (rs2479409; *r*^2^=4.4 × 10^−3^) was not associated with LDL-C in our study (*P*=0.72). Similarly, the *LDLR* p.Arg257Trp is not linked (*r*^2^=1.7 × 10^−4^) with the non-significant index SNP (rs6511720). The two single-variant associations identified in the current study may, by far, represent some of the strongest effect sizes (*β*=−0.64 s.d. for *PCSK9* p.Arg93Cys and *β*=0.91 for *LDLR* p.Arg257Trp) for any missense variant known to associate with plasma lipid levels. Interestingly, the association of *PCSK9* p.Arg93Cys was more prominent in PUUMA-MI (MAF=0.017; *β*=−0.65, *P*=3.2 × 10^−25^) compared with HKU-TRS (MAF=0.008; *β*=−0.61, *P*=3.7 × 10^−8^). The large discrepancy was mainly a result of the intra-population difference, in which the risk allele frequency of Northern Chinese is at least twice the frequency of Southern Chinese. Such a phenomenon was also observed for other rare variants, which underscores the need for rare variant association analysis in a more fine-scale local population.

### Novel loci identified at PKD1L3 for LDL-C and PNPLA3 for TG

**Figure 1:**
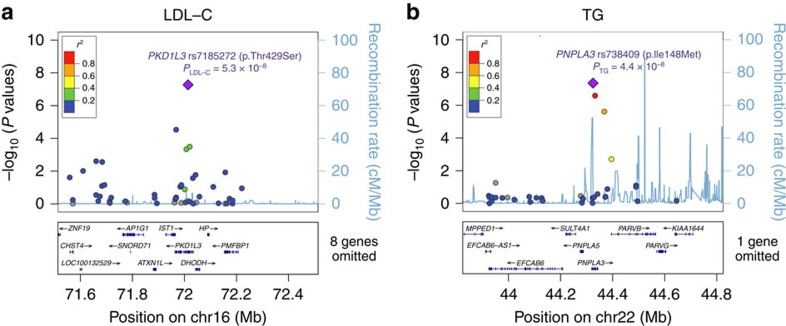
Regional plots of two newly discovered exome-wide significant associations. (**a**) *PKD1L3* rs7185272 with LDL-C and (**b**) *PNPLA3* rs738409 with TG. SNPs are coloured on the basis of their pairwise LD values (*r*^2^) with the top SNP (purple), which has the smallest *P* value in the region. Pairwise LD and the fine-scale recombination rate (light blue line) were estimated based on 1000 Genomes project (March 2012) ASN genotypes. SNPs not present in the reference panel are coloured in grey. Genes are presented by blue lines with arrows indicating the direction of transcription and rectangles as exons in the bottom panel.

Next, we sought to identify novel associations of plausibly functional variants. Along with the 16 known loci, 2 common nonsynonymous variants in genes/loci not previously implicated attained exome-wide significance (Table 1; Supplementary Fig. 2). Regional plots of associations for these two newly identified loci are illustrated in Fig. 1.

Here *PNPLA3* p.Ile148Met (rs738409), a missense variant known to associate with nonalcoholic fatty liver disease (NAFLD)^19^, was shown to strongly influence TG level (*β*=−0.072, *P*=4.4 × 10^−8^). As NAFLD is a condition tightly linked to obesity and type 2 diabetes mellitus (T2DM)^20^, we looked for factors that might confound the observed association. Particularly, the HKU-TRS cohort was overrepresented with T2DM patients relative to the general population. We therefore stratified the sample by T2DM status and tested if this enrichment contributed to the TG-lowering effect. Although stronger association was observed in T2DM cases (*N*=3,366; *β*=−0.114, *P*=8.4 × 10^−6^) versus controls (*N*=1,775; *β*=−0.067, *P*=0.032), no significant interaction between the additive effect and T2DM was detected (*P*=0.27). The effect size observed in the non-T2DM group was very similar to PUUMA-MI (*β*=−0.058, *P*=7.3 × 10^−4^), where no enrichment of T2DM cases was observed. Likewise, association of *PNPLA3* remained significant (conditional *P*=1.9 × 10^−5^ in HKU-TRS) after adjusting for body mass index, demonstrating a novel association independent of potential confounding factors.

The second strongest novel association mapped to a LD block encompassing the known locus of haptoglobin-related protein (*HPR*) at 16q22.2. Carriers of the *PKD1L3* minor allele (rs7185272, encoding p.Thr429Ser) had significantly lower LDL-C (*P*=5.4 × 10^−8^) and TC levels (*P*=2.5 × 10^−7^). This coding variant is not in LD with the GWAS SNP at nearby *HPR* (rs2000999, *r*^2^=0.027; *P*_LDL-C_=1.1 × 10^−3^, *P*_TC_=1.9 × 10^−3^), implying an independent signal of association (Supplementary Data 1). Another probably damaging, missense variant, rs1559401 encoding PKD1L3 p.His571Gln, is indeed in complete LD with *PKD1L3* p.Thr429Ser and had indistinguishable effect sizes on both lipid traits (*P*_LDL-C_=5.9 × 10^−8^; *P*_TC_=2.1 × 10^−7^). We further characterized the novel LDL-C lead SNP (rs7185272) by examining its association in other data sets. *In silico* look up in the largest publically available GWAS analyses of GLGC^4^ and CardioGRAM^21^ revealed equally strong associations with LDL-C (*P*=2.8 × 10^−9^) and TC (*P*=3.2 × 10^−10^) and slight though insignificant decrease in CAD risk (*P*=0.058).

### Gene-based test identified TEAD2 as new HDL-associated gene

Compared with common or low frequency variants, the statistical power of single-variant test to detect association of individual rare variant is generally limited. To improve power for identifying novel lipid-associated genes, we proceeded to a gene-based association test using RAREMETAL^22^, to assess the aggregate effect of rare variants across genes. Specifically, we restricted the analysis to nonsense variants and missense variants predicted to be damaging^23^. Two MAF thresholds (<1% and <5%) were employed for the (1) unweighted combined multivariate collapsing burden test (CMC)^24^ and (2) sequence kernel association test (SKAT)^25^, whereas only MAF<5% threshold was considered for the (3) variable-threshold test (VT)^26^.

Three known genes—*CETP* (*P*_HDL-C_=9.0 × 10^−35^)*, PCSK9* (*P*_LDL-C_=1.4−10^−34^; *P*_TC_=5.1 × 10^−27^) and *APOE* (*P*_LDL-C_=2.2 × 10^−34^; *P*_TC_=2.0 × 10^−12^)—exhibited strong association in gene-level tests with HDL-C, LDL-C and/or TC (Table 2). Except *APOE*, the gene-based *P* values exceeded the minimal *P* value among all single-variant tests; however, the associations were largely attenuated (*P*>0.01) while conditioning on the most significant missense variant, indicating that the signals were largely driven by a single coding SNP.

More importantly, via CMC burden test, we identified a significant gene-based association with HDL-C at a novel gene, *TEAD2* (*β*=1.11, *P*=1.9 × 10^−7^). Though two rare, missense variants (rs142665148, encoding p.Asp12Asn, MAC=1 and rs139131757, encoding p.Ala266Val, MAF=0.08%) predicted to be damaging were included in the *TEAD2* gene set, the gene-based association was mainly driven by the near exome-wide significant *TEAD2* p.Asp12Asn SNP (*β*=1.11, *P*=3.8 × 10^−7^). This missense variant was consistently found to increase HDL-C levels in both PUUMA-MI (*β*=1.16, *P*=9.7 × 10^−3^) and HKU-TRS (*β*=1.09, *P*=1.2 × 10^−5^). Another singleton missense SNP (*TEAD2* p.Ala266Val) also showed a consistent, though non-significant, HDL-C-increasing effect (*β*=1.15, *P*=0.25).

### Exome-wide significant variants were associated with CAD

**Figure 2:**
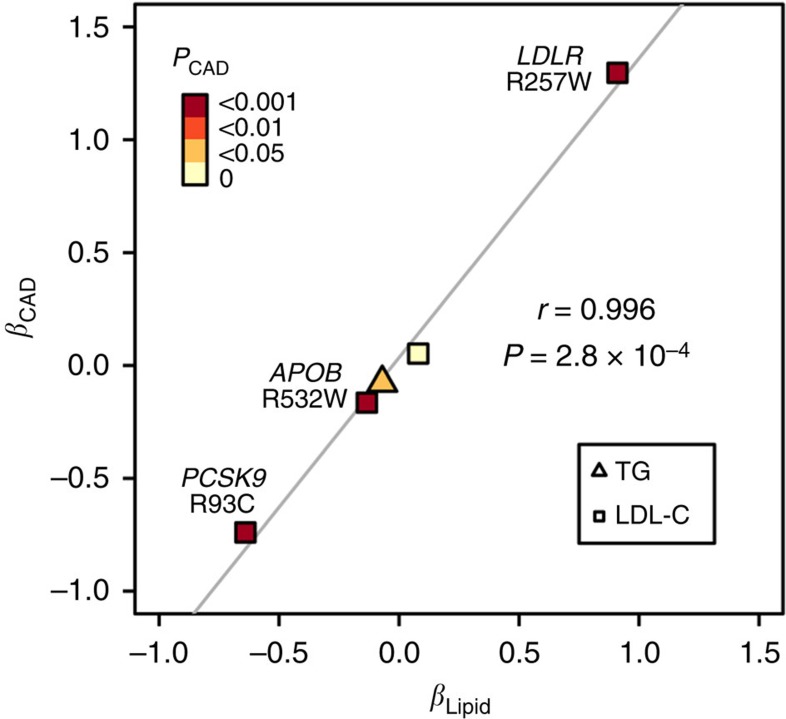
Comparison of effect sizes (*β*) between CAD and LDL-C (square) as well as TG (triangle) for protein-altering variants with independent association. Five missense variants denoted in Table 3 are illustrated. Points are coloured by the significance of association with CAD. Pearson's correlation coefficient (*r*) and line of best fit are shown.

We further explored the relationship between the lipid-associated loci and cardiovascular disease in the Chinese population. Specially, we tested whether the newly identified protein-altering variants showing independent associations with lipid traits also influenced CAD risk (Table 3). Among the seven variants tested, five SNPs showed a consistent direction of effect between lipid traits and CAD (*P*<0.05). In particular, the (or near) Asian-specific variants associated with LDL-C were also strongly associated with CAD. The LDL-decreasing *PCSK9* R93C (OR=0.48, *P*=3.8 × 10^−7^) and *APOB* R532W (OR=0.85, *P*=2.9 × 10^−4^) significantly protected against CAD, whereas the risk for CAD was 3.66 times higher for subjects carrying the LDL-increasing *LDLR* R257W allele (*P*=1.1 × 10^−4^). Compared with the common variants in the same region, these rare variants conferred much larger protective or damaging effects to CAD. In fact, we observed a strong positive correlation between the strengths of effect on plasma lipid levels and CAD risk (Fig. 2), highlighting the clinical relevance of rare variants with large effect in personalized medicine.

## Discussion

This study is the first Asian exome-wide association analysis on blood lipid levels. By genotyping 12,685 Chinese individuals using a custom exome array, we identified significant association at 19 loci. Three of these loci harbour lipid-associated missense variants, implicating three novel genes—*PNPLA3*, *PKD1L3* and *TEAD2—*not previously described. For the other 16 known loci, we not only confirmed the association signals of known GWAS hits, but also identified novel independent associations and, above all, revealed 3 Asian-specific associations involving rare nonsynonymous changes. This has important implications in study design, such that, by examining the less studied non-European populations, novel associations can be found even with a relatively small sample size.

A major finding of our current study is the discovery of three novel lipid-associated loci. The probably damaging, missense variant encoding PNPLA3 p.Ile148Met, a TG lipase, was previously demonstrated to show strong association with NAFLD^27^,^28^,^29^. NAFLD refers to a wide spectrum of chronic liver disorders, ranging from hepatic steatosis to nonalcoholic steatohepatitis. It is characterized by increased hepatic fat content and hepatic TG level, and is tightly linked with signatures of cardiovascular diseases such as obesity, insulin resistance and T2DM^30^. The nonsynonymous change was demonstrated to reduce TG-rich very low density lipoprotein efflux, limit substrate-binding to the nearby catalytic residue and thereby impair hydrolase activity^31^,^32^. While *PNPLA3* has been the most robustly replicated locus for NAFLD^20^, its association with plasma TG levels remains inconclusive^27^,^33^,^34^. No significant association was reported in population-based studies; however, the minor allele G (encoding methionine) was associated with lower TG levels in extremely obese individuals and NAFLD patients^35^,^36^,^37^. Although these studies have shown suggestive association with lipid variation, our current study was first to demonstrate a genome-wide significant association with TG. Multiple lines of evidence from animal studies suggest that the catalytic inactivation of PNPLA3 can be exacerbated by overexpressing p.Ile148Met under dietary conditions, which expose the liver to high levels of insulin^33^. Moreover, insulin increased *PNPLA3* transcription through LXR-mediated activation of SREBP-1c^36^, in line with the increased *PNPLA3* mRNA levels observed in obese, insulin-resistant animals^38^. These observations may provide an explanation for the slightly stronger effect of *PNPLA3* p.Ile148Met with TG in the T2DM patients observed in HKU-TRS.

*PKD1L3*, encoding an ion channel of the polycystic kidney disease-like family, is located at ∼100 kb away from a known LDL-cholesterol locus of haptoglobin-related protein (*HPR*)^39^. Although associated variants in both genes increase levels of LDL-C and TC, we have demonstrated in our study that their associations are highly independent. Besides the probably damaging TC lead SNP, its LD proxy (rs7185272), also the lead SNP for LDL-C, maps to a region with transcriptional factor-binding signals for *TCF7L2* and *FOXA2*/*FOXA1* in the ENCODE Consortium ChIP-seq data^40^*. TCF7L2* is a well-known type 2 DM gene affecting both fasting glucose and fasting insulin levels^41^,^42^,^43^. Likewise, *FOXA2* regulates expression of genes essential for maintaining glucose homeostasis^41^,^44^. Indeed, *PKD1L3* was implicated as a putative sour taste receptor^39^ and its expression is most abundant in liver^45^. It is tempting to speculate that *PKD1L3* is directly involved in glucose and lipid metabolism. The rs7185272 variation might disrupt binding of TCF7L2 or FOXA2, perturbing the expression of *PKD1L3* and the regulation of plasma LDL-C levels.

*TEAD2*, also known as transcriptional enhancer factor 4 (TEF-4), was identified as a HDL-associated gene through gene-based burden test. It is involved in the pathway of peroxisome proliferator-activated receptor (PPARα) regulation on lipid metabolism, according to the Reactome database^46^. TEF-4 also functions as a transcription factor for CTP:phosphocholine cytidylyltransferase α, which is involved in biosynthesis of phospholipid. Concentration of phospholipid, being the integral component of the hydrophilic coat of HDL-C^47^, has been suggested to correlate with HDL concentration by possibly modulating the cellular cholesterol efflux^48^.

Consistent with other fine-mapping and exome-wide genetic analyses^18^,^49^, we detected multiple independent association signals contributed by missense changes in known lipid-associated genes. Specially, we identified associations of two missense changes (*APOB* R532W and *APOA5* G185S) with very low frequency in non-Asian populations, as well as three Asian-specific variants (*CETP* D459G, *PSCK9* R93C and *LDLR* R257W). In line with their large effects on lipid levels, all of these population-specific rare variants that influence LDL-C have significantly large impact on CAD risk. The 50% reduction in CAD risk for *PCSK9* and the threefold increase for *LDLR* rare variants are indeed unprecedented in GWAS of such a complex disorder. Further work is required to validate these associations in a large-scale study of an Asian cohort and to determine the causal effect.

One of the potential limitations of our study was that the coverage of rare variants on the exome array was suboptimal in the Chinese population. While most of the rare functional variants are private to specific populations, under-representation of the causative rare variants might limit statistical power, particularly for gene-based burden tests. Exome or whole genome sequencing is therefore needed to fully characterize and capture these population-specific variants. We attempted to ameliorate this deficiency by adding 30,368 additional coding variants to the array, increasing the number of polymorphic coding variants (77,073 and 90,486 in HKU-TRS and PUUMA-MI, respectively) to approach the number observed in Europeans (80,137 in HUNT-MI).

In summary, missense variants of three novel loci—*PNPLA3*, *PKD1L3* and *TEAD2*—showed strong evidence of association with blood lipid levels, alongside with three Asian-specific association signals in known loci (*CETP, PSCK9* and *LDLR)*. Understanding the genetic architecture of how these deleterious rare and regulatory common variants together affect disease risk could provide more insights into the causal mechanisms, and also facilitate the translation of these genetic findings to drug discovery and personalized medicine.

## Methods

### Subjects and phenotypes

Our study comprised two main cohorts, HKU-TRS and PUUMA-MI, with a total of 12,685 study participants of Chinese ancestry genotyped and passed quality control. Study protocols were approved by the institutional review boards of all institutions involved in the study and written informed consent was obtained from all study participants.

*HKU-TRS*. We genotyped 6,048 Southern Chinese subjects recruited from the Chinese CAD Cohort of the Queen Mary Hospital in Hong Kong; Hong Kong Cardiovascular Risk Factor Prevalence Study (CRISPS)^50^ and Hong Kong West Diabetes Registry (HKWDR)^51^. Detailed descriptions of the corresponding cohorts are provided in Supplementary Note 1.

Fasting venous blood samples were collected for DNA and laboratory-based biochemical analyses. General physical measurements and medical and drug histories of each subject were recorded. Plasma lipids were measured by standard enzymatic methods^50^,^51^. LDL-C level was calculated using the Friedewald equation^52^ or by direct enzymatic colorimetric test if TG was >4.5 mmol l^−1^. Extreme lipid levels (>4 s.d. from the mean) were set as missing to avoid false positive findings due to outliers. For the quantitative blood lipids analyses, we included only 5,233 subjects who were not taking any lipid-lowering drug or those with their pre-treatment lipid levels available. Clinical characteristics of subjects involved in the lipid association analysis are shown in Supplementary Table 9.

To evaluate if the lipid-associated loci also influence CAD, 2,372 CAD cases and 3,388 non-CAD controls were included in the data set (Supplementary Table 10). CAD cases were defined as having coronary artery revascularization interventions (including percutaneous coronary intervention and/or coronary artery bypass graft surgery); or those who had been diagnosed with MI; or those with stenosis of >50% in one or more of the major epicardial coronary arteries by angiogram. Subjects who had no documented history or angiogram evidence of CAD were defined as non-CAD controls. Written informed consents were obtained from all subjects, and the study protocol was approved by the Institutional Review Board of the University of Hong Kong/Hospital Authority Hong Kong West Cluster.

*PUUMA-MI*. PUUMA-MI is a large-scale project designed to study cardiovascular disease and related traits, including MI and plasma lipid levels, in China. Samples were collected by the Joint Institute of the PUUMA-MI. In total, 8,621 samples from 4 hospitals were collected for genotyping: 5,606 from Peking University First Hospital, 1,209 from Peking University Third Hospital, 774 from Beijing Shijingshan Hospital and 482 from Asia Heart Disease Hospital of Wuhan.

Fasting plasma lipid levels were measured for Peking University First Hospital-based samples and Beijing Shijingshan cohort after overnight fasting. For samples collected from Peking University Third Hospital, TC and TG were measured by an enzymatic method while HDL-C and LDL-C were measured by a liquid selective detergent method. Details of the cohort description are provided in Supplementary Note 1 and Supplementary Table 11.

According to the 2004 American College of Cardiology/American Heart Association guideline^53^, criteria for acute MI are detection of rise and/or fall of cardiac biomarkers (preferably troponin) with at least one value above the 99th percentile of the upper reference limit together with evidence of myocardial ischaemia with at least one of the following: symptoms of ischaemia; ECG changes indicative of new ischaemia (new ST-T changes or new left bundle branch block); development of pathological Q waves in the ECG; imaging evidence of new loss of viable myocardium or new regional wall motion abnormality. Any one of the following criteria meets the diagnosis for prior MI: development of new pathological Q waves with or without symptoms; imaging evidence of a region of loss of viable myocardium that is thinned and fails to contract, in the absence of a non-ischaemic cause. For other individuals, self-reported CAD status was collected as well at the time of blood draw. In total, we have 550 MI cases and 5,606 non-CAD controls from Peking University First Hospital, 876 MI cases and 333 non-CAD controls from Peking University Third Hospital, 256 MI cases and 226 non-CAD controls from Wuhan Asia Heart Hospital, and 774 non-CAD controls from Beijing Shijingshan Hospital.

*HUNT-MI*. A total of 5,643 Norwegians were selected from the second survey of the HUNT, including 2,349 medical-record confirmed MI cases and 2,317. Details of the Norwegian sample, genotyping and phenotypes have been described^18^.

### Genotyping

All subjects were genotyped using the Asian Exomechip, a specially designed exome array with a custom content of 58,317 variants on top of the standard Infinium HumanExome BeadChip (Illumina, CA), which interrogated a total of 302,218 variants. Details of the Asian Exomechip design has been described in a number of studies, including the breast cancer exome-wide association analysis on Chineses^17^. In brief, the original design of the exome array includes 242,901 markers, with the majority of over 200K coding variants identified from ∼12,000 sequenced genomes and exomes of primarily European ancestry. The underrepresentation of non-European genomes in the original design limited the coverage of low frequency variants in Asian populations^54^. To allow the comprehensive genotyping across the full allele frequency spectrum, a custom panel of ∼30K nonsense/missense variants were added based on 3 independent Asian sequencing data sets of ∼1,000 Chinese samples. Integrated in the custom panel were also common variants selected for GWAS follow-up or fine mapping studies. Additional information regarding the design is provided in Supplementary Methods. Genotype calling was performed using GenTrain version 2.0 in GenomeStudio V2011.1 (Illumina) independently for both cohorts, followed by cohort-specific quality control.

### Data quality controls

*HKU-TRS*. Manual inspection of genotype clusters was first carried out for >55,000 variants that either (1) showed evidence of bad genotype clustering in exome array genotyping of over 9,000 subjects by collaborators^17^,^55^, or (2) had GenTrain score <0.8 or (3) had high missingness (>1%). A total of 4,550 markers were removed due to poor genotype clustering. Indiviudal-level QC was carried out with regard to duplication, gender mismatch, possible sample contamination and biological relatedness. We further employed a variant-level QC that removed SNPs with >2% missingness or violated Hardy–Weinberg equilibrium (*P*<1 × 10^−5^), or SNPs originally designed with the purpose of quality control. Details regarding the quality controls are given in the Supplementary Methods. After all quality control measures, 5,233 samples and 286,795 SNPs, of which 176,149 variants were monomorphic, remained in the data set and were subject to association analysis.

*PUUMA-MI*. To obtain high quality genotypes, strict criteria were applied to filter out low quality genotypes. We undertook plate-, individual- and variant-level checks to exclude poor quality genotype calls from the data set (Supplementary Methods). Briefly, the individual-based QC criteria for filtering included a call rate of <99%, gender mismatch, excess heterozygosity and relatedness. Variant-level QC was performed to exclude variants with low cluster score, low call rate (<99.9%) and those deviated from Hardy–Weinberg equilibrium (*P*<1 × 10^−4^). Finally, 7,452 samples and 282,456 markers were retained after quality control. Among the successfully genotyped markers, 129,306 are polymorphic in the Chinese samples. To further verify marker allele assignment, we examined the correlation between allele frequency of the PUUMA-MI Chinese samples and those of East Asian samples from 1000 Genomes Project, and a high correlation (*r*^2^=0.98) was observed.

### Phenotype transformation

For the HKU-TRS data set, log transformation was first performed on TG and HDL-C. Phenotypes were then defined as the standardized residuals of blood lipid levels after adjustment on age, gender and two prinicpal components computed from ∼22K common SNPs (MAF>5%). For the PUUMA-MI data, blood lipid levels were transformed to rank-based inverse normal residuals to minimize the impact of outliers and ensure normality. Age, sex, MI status (case or control) and 10 principal components estimated from AIMs were included as covariates in the association tests.

### Meta-analysis for lipid traits

We then proceeded with meta-analyses of both single-variant and gene-based association tests using a framework of rvtests coupled with RAREMETAL. First, in each cohort, we carried out single-variant association tests for all markers passing quality controls using rvtests (http://zhanxw.github.io/rvtests/). The computed score test statistics as well as the corresponding variance-covariance matrix, which summarizes the LD between markers, were shared between studies. Overall, 145,276 SNPs were polymorphic in at least 1 individual in the combined data set.

Next, we performed single-variant meta-analysis by combining the score test results using RAREMETAL. We restricted the analysis to SNPs that were polymorphic in both studies and had at least 20 minor alleles in the combined data set (MAC>=20). Exome-wide significance threshold was defined as 2.69 × 10^−7^, which accounted for multiple testing of 65,671 SNPs passing the filtering criteria (see next section). The test statistics, as visualized in a quantile–quantile plot, appeared well-calibrated (Supplementary Fig. 10).

To leverage power for detecting low frequency and rare variants, we performed gene-based meta-analyses, including a CMC^24^, VT^26^ and SKAT^25^, to evaluate their aggregate effects in each gene. We only included loss-of-function (stopgain and splicing) and missense variants predicted to be damaging into the gene sets. Damaging variants were predicted by KGGseq^23^, which combined multiple functional prediction methods (for example, SIFT, PolyPhen2 and CADD) to predict pathogenic variants. Two MAF thresholds (<1% and <5%) were employed for CMC and SKAT, whereas only MAF<5% threshold was considered for VT. Genes with at least 20 copies of rare alleles were considered (*n*=8,369 for MAF<1% and 9,025 for MAF<5%). Gene-based significance threshold was defined as *P*<8.43 × 10^−7^, which was equivalent to the multiple testing correction of 59,313 effective number of tests (see next section).

### Meta-analysis for *CAD*

Logistic regression was carried out in HKU-TRS and PUUMA-MI to evaluate if the newly identified lipid-associated SNPs influence CAD risk. The effect estimates and s.e. were meta-analysed using METAL by the fixed-effect inverse-variance method. Considering multiple testing of seven association tests, we defined SNPs with *P*<7.1 × 10^−3^ as being associated with CAD.

### Identification of independent signals

To delineate SNPs with independent association, we performed stepwise conditional analysis using the known lipid-associated variants within 500 kb of the lead SNPs as covariates by RAREMETAL. For each locus, SNPs with conditional *P* values reaching exome-wide significance (*P*<2.69 × 10^−7^) were considered as having independent association and the lead SNP showing strongest evidence of independent association was later included in the regression model as a covariate. The process was repeated until no variant reached exome-wide significance after adjustment.

### Estimating the effective number of independent tests

While the 4 blood lipid traits are highly correlated and there existed LD among common and low-frequency variants, traditional Bonferroni correction for multiple testing of all 262,684 correlated single-variant tests (*P*<1.90 × 10^−7^) was likely to be too stringent, leading to an overcorrection of the association. To take into account of the dependences, we estimated the effective number of independent tests (*M*_e_) for both phenotypes and genotypes using methods based on eigenvalues of the corresponding correlation matrix^56^,^57^. We estimated the *M*_e_ to be 3.41 for phenotypes and 54,463 for genotypes, totalling 185,719 independent single-variant tests. Exome-wide significance threshold for single-variant meta-analysis was therefore defined as 2.69 × 10^−7^ (=0.05/185,719). Similarly, for gene-based meta-analysis, exome-wide significance threshold was defined as *P*=8.43 × 10^−7^, equivalent to multiple testing correction of 59,313≅(8,369+9,025) × 3.41 independent tests for the 8,369 and 9,025 genes with MAF <1% and <5%, respectively.

### Asian-specific variants

According to the ancestry of samples (http://www.1000genomes.org), we divided the individuals of 1000 Genomes into three populations: Asian (CHB, CHS and JPT), European (CEU, FIN, GBR, IBS and TSI) and African (ASW, LWK and YRI). For each population, the MAF was estimated separately. Variants were classified as Asian-specific if they were polymorphic (MAF>0) in the Asian population but monomorphic in both the European and African populations.

## Additional information

**How to cite this article**: Tang, C. S. *et al*. Exome-wide association analysis reveals novel coding sequence variants associated with lipid traits in Chinese. *Nat. Commun.* 6:10206 doi: 10.1038/ncomms10206 (2015).

## Supplementary Material

Supplementary Information Supplementary Figures 1-10, Supplementary Tables 1-11, Supplementary Note 1, Supplementary Methods and Supplementary References

Supplementary Data 1 Conditional analysis for all loci attaining exome-wide significance

## Figures and Tables

**Table 1:** Variants independently associated with blood lipids at exome-wide significance (*P*<2.69 × 10^−7^).

**Locus**	**SNPs**	**Annotation**	**Position**	Alleles*	**Trait(s)**	**PUUMA-MI (*****N*****=7,452)**			**HKU-TRS (*****N*****=5,233)**			**Combined (*****N*****=12,685)**		
						**EAF (%)**†	**Effect (s.e.)**‡	***P***	**EAF (%)**	**Effect (s.e.)**	***P***	**EAF (%)**	**Effect (s.e.)**	***P***
*Novel associations at newly identified loci*														
* PKD1L3*	rs7185272	p.Thr429Ser	16:72013797	G/C	LDL-C	76.0	0.07 (0.02)	6.4 × 10^−4^	71.1	0.09 (0.02)	1.4 × 10^−5^	74.0	0.08 (0.01)	5.4 × 10^−8^
					TC		0.06 (0.02)	1.5 × 10^−3^		0.09 (0.02)	2.7 × 10^−5^		0.07 (0.01)	2.5 × 10^−7^
* PNPLA3*	rs738409	p.Ile148Met	22:44324727	C/G	TG	36.5	−0.06 (0.02)	7.3 × 10^−4^	37.1	−0.09 (0.02)	6.9 × 10^−6^	36.7	−0.07 (0.01)	4.4 × 10^−8^
*Asian-specific associations at known loci*														
* PCSK9*	rs151193009	p.Arg93Cys	1:55509585	C/T	LDL-C	1.7	−0.65 (0.06)	3.2 × 10^−25^	0.8	−0.61 (0.11)	3.7 × 10^−8^	1.3	−0.64 (0.05)	7.9 × 10^−32^
					TC		−0.58 (0.06)	2.0 × 10^−20^		−0.50 (0.11)	4.0 × 10^−6^		−0.56 (0.05)	5.1 × 10^−25^
* CETP*	rs2303790	p.Asp459Gly	16:5701729	A/G	HDL-C	2.3	0.36 (0.05)	3.3 × 10^−11^	3.2	0.53 (0.06)	1.5 × 10^−20^	2.7	0.44 (0.04)	3.2 × 10^−29^
* LDLR*	rs200990725	p.Arg257Trp	19:11217315	C/T	LDL-C	0.03	1.43 (0.45)	1.5 × 10^−3^	0.3	0.83 (0.18)	2.6 × 10^−6^	0.1	0.91 (0.17)	3.0 × 10^−8^
*Other independent associations at known loci*														
* APOB*	rs13306194	p.Arg532Trp	2:21252534	G/A	LDL-C	11.9	−0.13 (0.03)	7.5 × 10^−8^	14.2	−0.13 (0.03)	3.6 × 10^−6^	12.8	−0.13 (0.02)	1.2 × 10^−12^
					TC		−0.12 (0.03)	1.0 × 10^−8^		−0.14 (0.03)	1.2 × 10^−6^		−0.13 (0.02)	6.0 × 10^−12^
* APOA5*§	rs10466588	Intergenic	11:116610249	A/G	TG	14.2	0.08 (0.02)	4.4 × 10^−4^	10.3	0.07 (0.03)	0.036	12.6	0.08 (0.02)	4.7 × 10^−5^
					HDL-C		−0.09 (0.02)	1.7 × 10^−4^		−0.03 (0.03)	0.321		−0.07 (0.02)	2.9 × 10^−4^
* APOA5*	rs2075291	p.Gly185Cys	11:116661392	C/A	HDL-C	6.0	−0.32 (0.03)	8.1 × 10^−21^	6.1	−0.27 (0.04)	1.4 × 10^−10^	6.0	−0.30 (0.03)	1.2 × 10^−29^
* DOCK6*	rs737337	p.Thr712Thr	19:11347493	T/C	TC	29.2	−0.10 (0.02)	5.5 × 10^−8^	24.6	−0.07 (0.02)	2.3 × 10^−3^	27.3	−0.09 (0.01)	7.5 × 10^−10^
* APOE*	rs445925	Intergenic	19:45415640	G/A	TC		−0.26 (0.03)	6.0 × 10^−18^		−0.20 (0.03)	4.8 × 10^−10^		−0.23 (0.02)	4.1 × 10^−26^
* APOE*	rs769449	Intronic	19:45410002	G/A	HDL-C	8.5	−0.18 (0.03)	5.2 × 10^−10^	7.6	−0.08 (0.04)	0.026	8.1	−0.14 (0.02)	3.8 × 10^−10^

EAF, Effect allele frequency; HDL-C, high-density lipoprotein cholesterol; HKU-TRS, Hong Kong Theme-based Research Scheme; LDL-C, low-density lipoprotein cholesterol; PUUMA-MI, Peking University Health Science Center and the University of Michigan Medical School study of Myocardial Infarction; SNP, single nucleotide polymorphism; TC, total cholesterol; TG, triglyceride.

For SNPs associated with more than one lipid traits, association results were listed first with primary trait, followed by secondary trait(s).

^*^Reference/alternative effect alleles with respect to human reference genome hg19.

^†^EAFs, in percentage, are shown for PUUMA-MI, HKU-TRS and the combined meta-analysis.

^‡^Effect sizes with respect to the effect allele are presented in s.d.

^§^SNP with significant association after conditioning on the known variant. For results of conditional analysis, see Supplementary Data 1.

**Table 2:** Genes significantly associated with lipid traits (*P*<8.43 × 10^−7^) in gene-based association test.

**Gene**	**Number of variants**	**CMAF(%)**	**Trait**	Best gene-based test*	**Effect**†	***P***
*CETP*	3	3.44	HDL-C	SKAT<5%	0.285	9.0 × 10^−35^
*PCSK9*	7	1.86	LDL-C	SKAT<5%	−0.472	1.4 × 10^−34^
			TC	SKAT<5%	−0.414	5.2 × 10^−27^
*APOE*	3	3.74	LDL-C	SKAT<5%	−0.408	2.2 × 10^−34^
			TC	burden<5%	−0.237	2.0 × 10^−12^
*TEAD2*	2	0.09	HDL-C	burden<1%	1.112	1.9 × 10^−7^

CMAF, cumulative minor allele frequency of all damaging variants included in the gene-based test, which gave the best *P* value; HDL-C, high-density lipoprotein cholesterol; LDL-C, low-density lipoprotein cholesterol; TC, total cholesterol.

^*^Gene-based tests (SKAT, VT or burden) for damaging and missense variants with <5% or <1% minor allele frequency.

^†^Effect of gene-based test is estimated from burden test (CMC) of the corresponding MAF cut-off threshold.

**Table 3:** CAD case–control association analysis for all protein-altering variants with independent association.

**Locus**	**SNPs**	**Alleles**	**Trait(s)**	**Annotation**	**Freq**	**PUUMA-MI (1,462 cases+5,983 controls)**		**HKU-TRS (2,372 cases+3,388 controls)**		**Combined (3,834 cases+9,371 controls)**	
					**(%)**	**OR (95%CI)**	***P***	**OR (95%CI)**	***P***	**OR (95%CI)**	***P***
*APOA5*	rs2075291	C/A	HDL-C	p.Gly185Cys	6.0	1.07 (0.89–1.29)	0.47	1.25 (1.06–1.46)	7.6 × 10^−3^	1.17 (1.03–1.32)	0.013
*CETP*	rs2303790	A/G	HDL-C	p.Asp459Gly	2.7	0.98 (0.72–1.32)	0.89	0.96 (0.77–1.20)	0.74	0.97 (0.81–1.16)	0.73
*LDLR*	rs200990725	C/T	LDLC	p.Arg257Trp	0.1	12.12 (1.46–100.33)	0.052	3.22 (1.62–6.41)	8.8 × 10^−4^	3.66 (1.90–7.04)	1.1 × 10^−4^
*PCSK9*	rs151193009	C/T	LDL-C, TC	p.Arg93Cys	1.3	0.48 (0.34–0.66)	2.1 × 10^−4^	0.47 (0.28–0.78)	3.7 × 10^−3^	0.48 (0.36–0.63)	3.8 × 10^−7^
*APOB*	rs13306194	G/A	LDL-C, TC	p.Arg532Trp	12.8	0.82 (0.71–0.93)	4.8 × 10^−3^	0.87 (0.77–0.97)	0.013	0.85 (0.78–0.93)	2.9 × 10^−4^
*PKD1L3*	rs7185272	G/C	LDL-C, TC	p.Thr429Ser	76	1.00 (0.91–1.12)	0.89	1.10 (1.01–1.20)	0.029	1.05 (0.99–1.12)	0.11
*PNPLA3*	rs738409	C/G	TG	p.Ile148Met	36.5	0.96 (0.88–1.05)	0.40	0.90 (0.83–0.97)	8.8 × 10^−3^	0.93 (0.87–0.98)	0.011

CAD, coronary artery disease; CI, confidence interval; HDL-C, high-density lipoprotein cholesterol; HKU-TRS, Hong Kong Theme-based Research Scheme; LDL-C, low-density lipoprotein cholesterol; OR, odds ratio; PUUMA-MI, Peking University Health Science Center and the University of Michigan Medical School study of Myocardial Infarction; SNP, single nucleotide polymorphism; TC, total cholesterol; TG, triglyceride.
