# Supplementary material for: Exome-wide association analysis reveals novel coding sequence variants associated with lipid traits in Chinese
Source: Nat Commun. 2015 Dec 22;6:10206. doi: 10.1038/ncomms10206 (PMC4703860; doi:10.1038/ncomms10206)
Supplement: Supplementary Information — Supplementary Figures 1-10, Supplementary Tables 1-11, Supplementary Note 1, Supplementary Methods and Supplementary References [file ncomms10206-s1.pdf]

Supplementary Figures

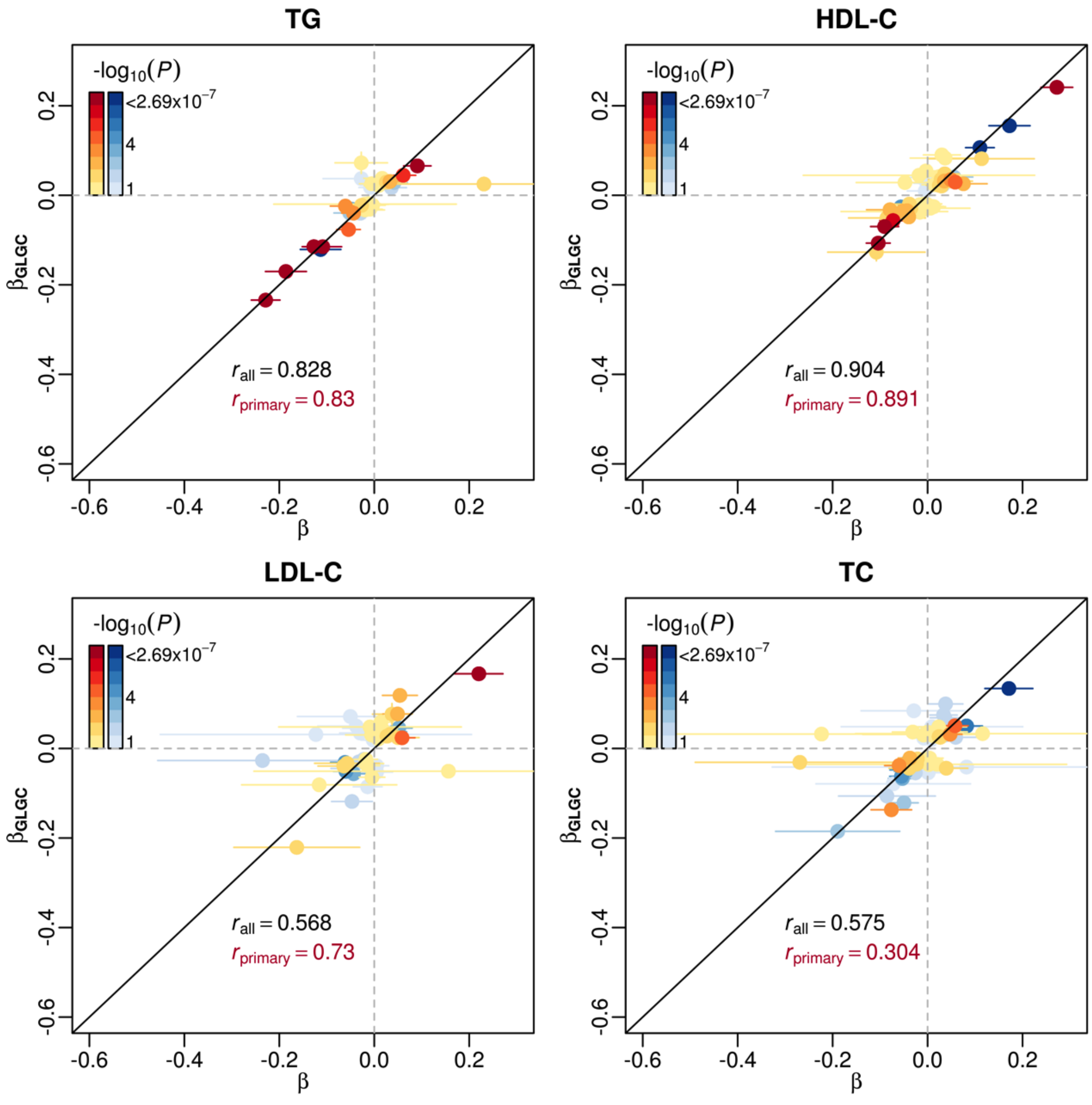

Supplementary Figure 1. Association evidence for genome-wide significant loci (n=157) reported in GLGC *et al.* (2013)[1]. Standardized effects (β, in s.d.) of primary (red) and secondary (blue) lipid traits estimated in GLGC on Europeans are plotted against estimates on Chinese. Error bars indicate the 95% confidence intervals. The theoretical lines of equality and Pearson correlation coefficients (*r*) for effects of primary and all traits are shown.

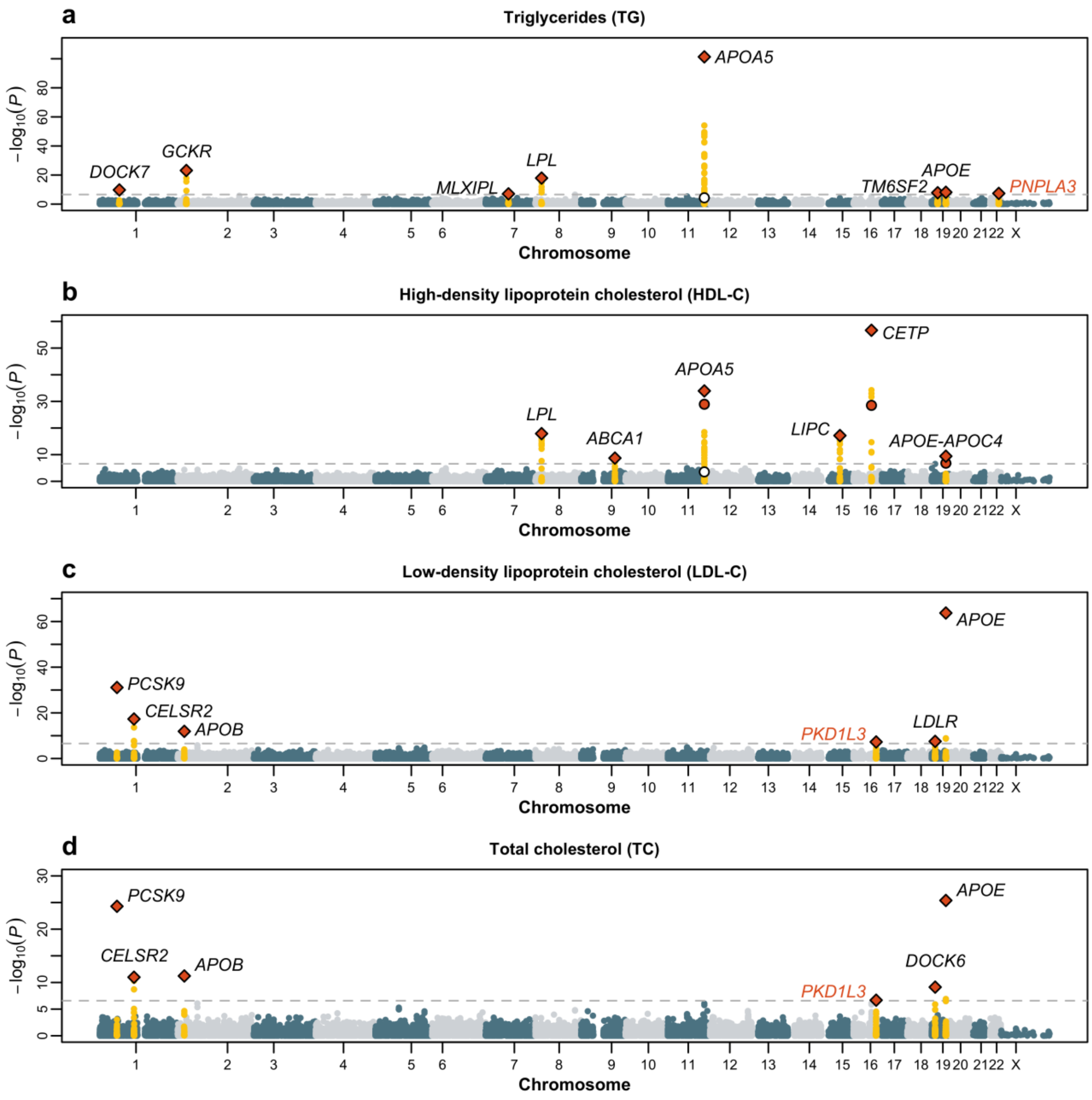

Supplementary Figure 2. Manhattan plot of association *P*-values for (a) TG, (b) HDL-C, (c) LDL-C and (d) TC. SNPs within loci (±1Mb of top SNP) passing exome-wide significance (horizontal dash line; $P<2.69 \times 10^{-7}$) are labelled in yellow and the top SNP of each region are labelled separately in orange diamond. SNPs showing independent association after conditional analysis are denoted by cicles.

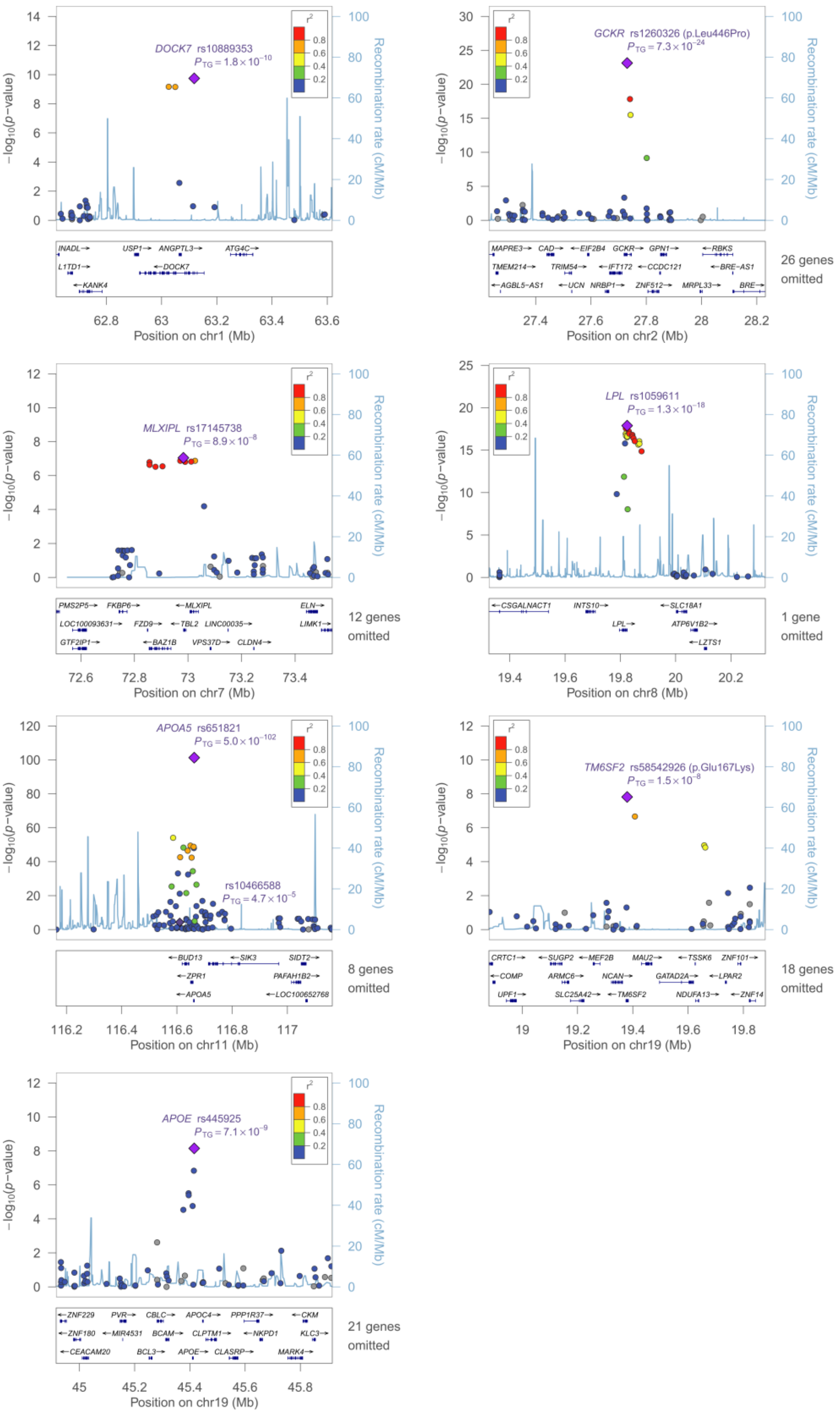

Supplementary Figure 3. Regional plots of known loci showing exome-wide significant association for triglycerides.

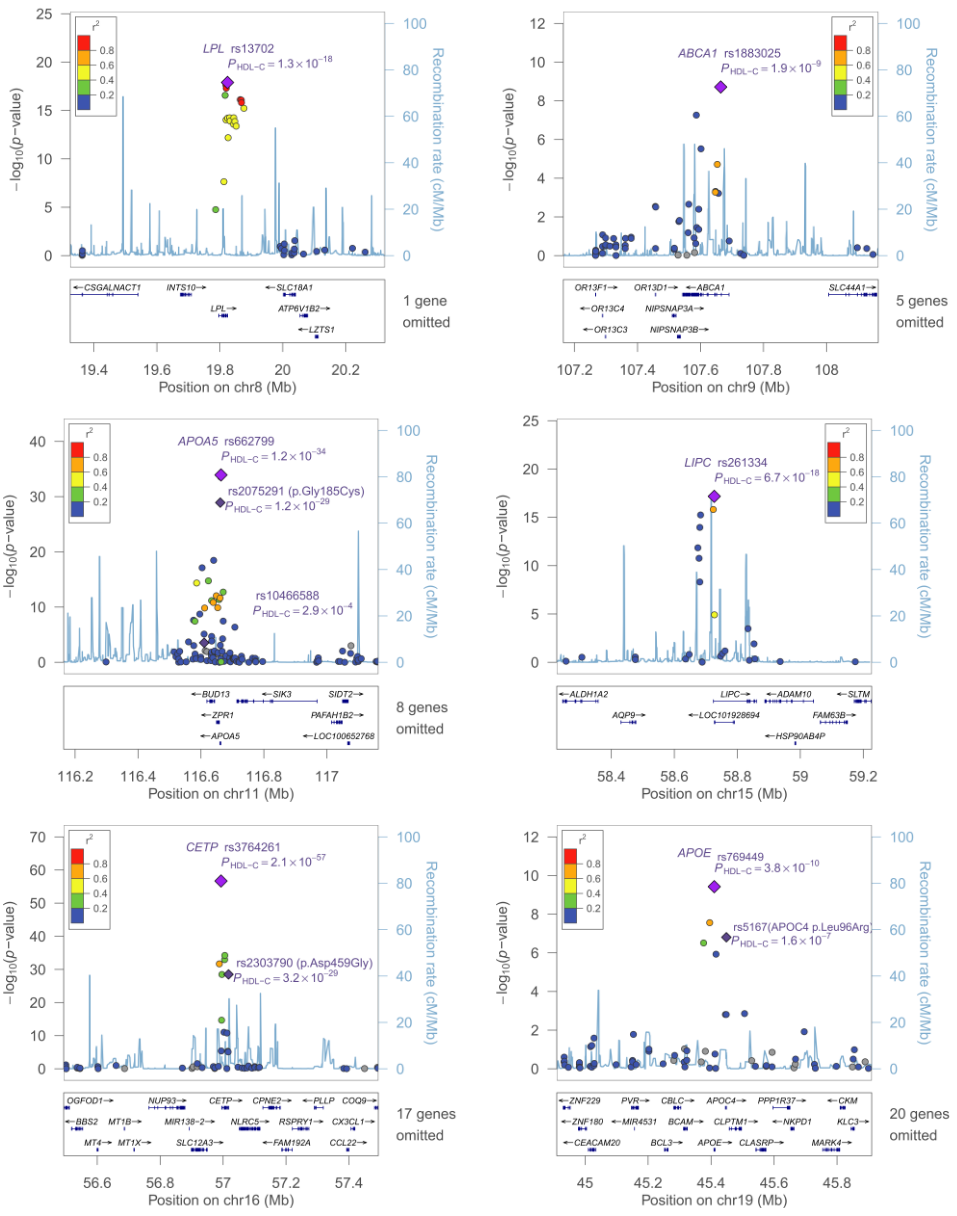

Supplementary Figure 4. Regional plots of known loci showing exome-wide significant association for HDL cholesterol.

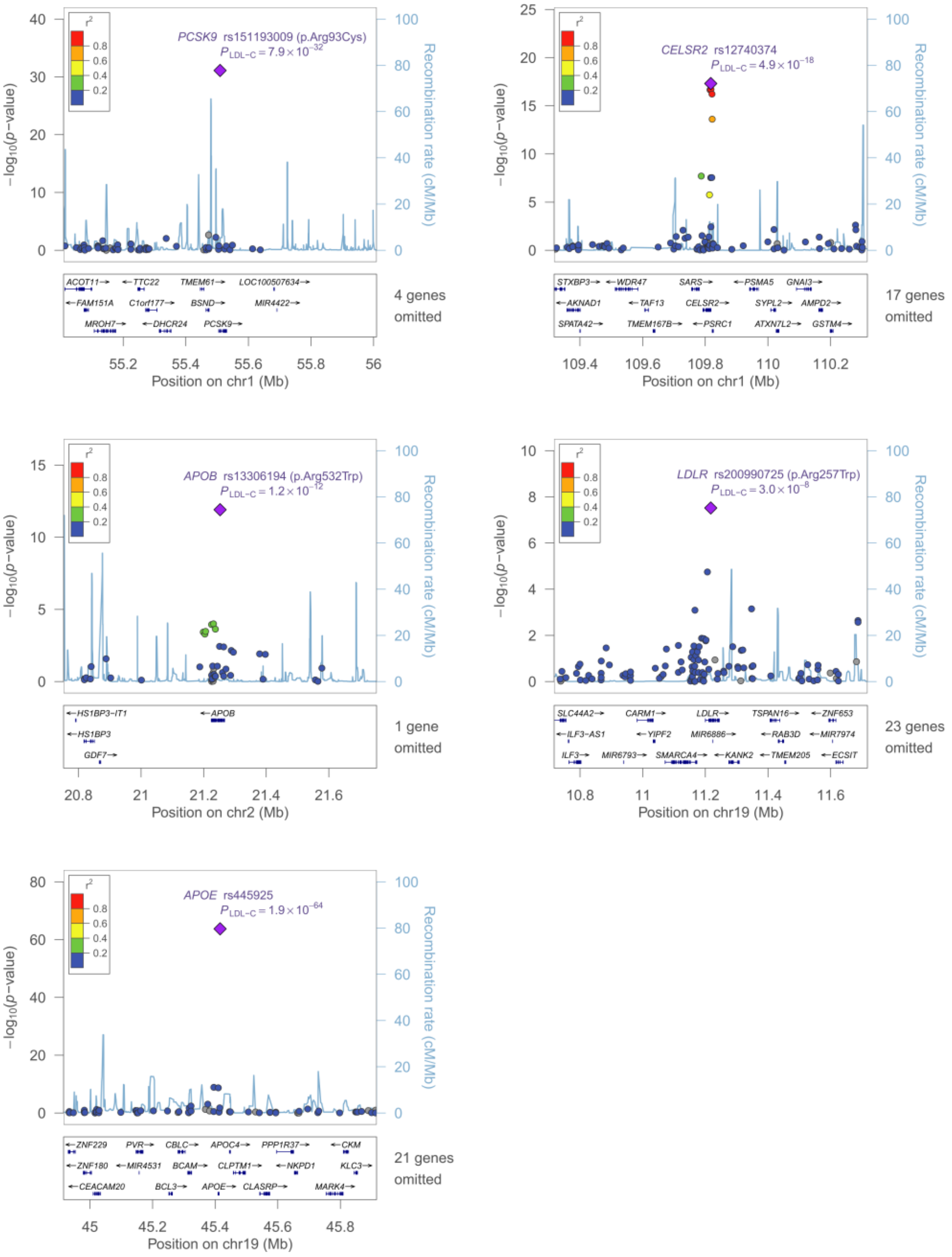

Supplementary Figure 5. Regional plots of known loci showing exome-wide significant association for LDL cholesterol.

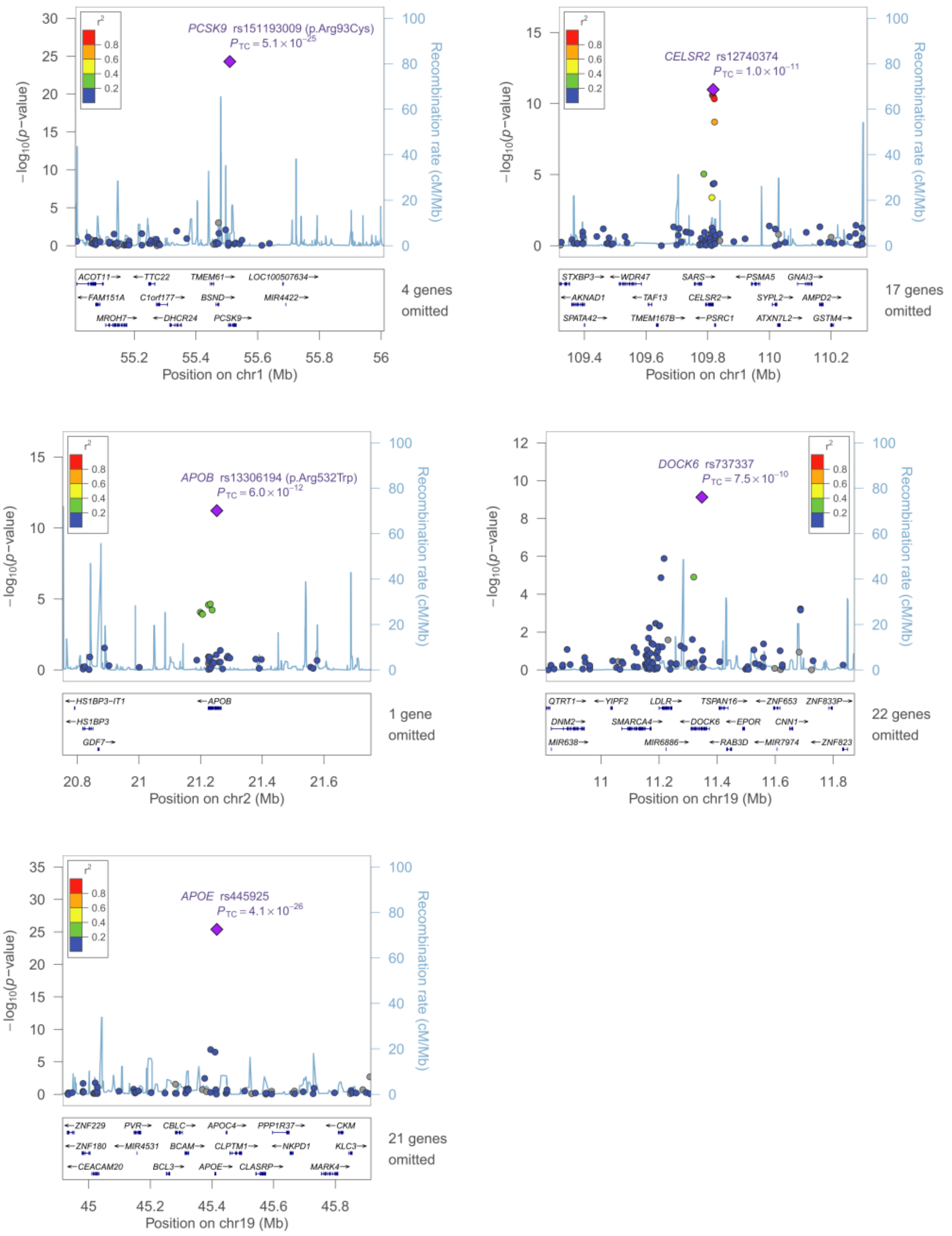

Supplementary Figure 6. Regional plots of known loci showing exome-wide significant association for total cholesterol.

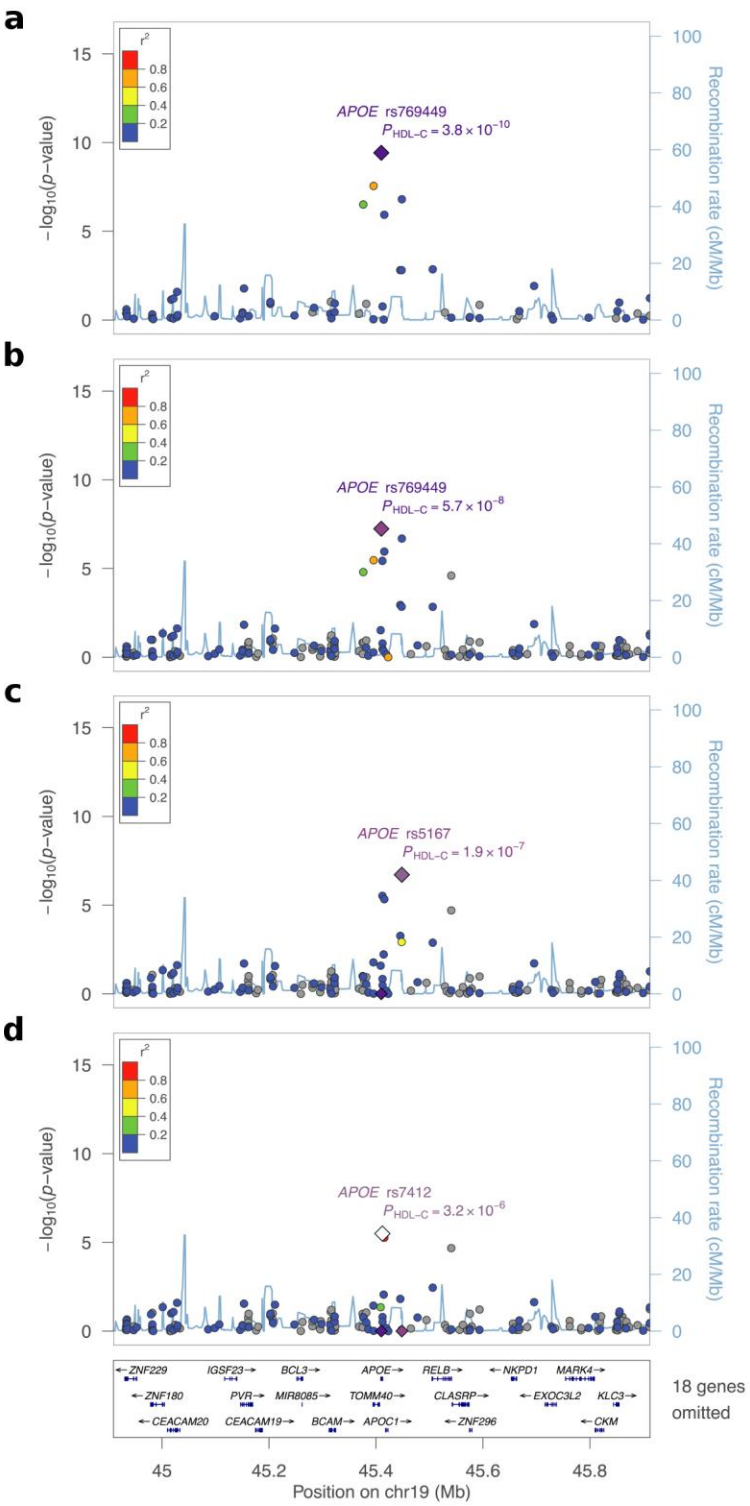

Supplementary Figure 7. Regional plots of conditional analysis between *APOE* and HDL cholesterol. Association *p*-values are shown (a) before and after stepwise conditioning on (b) known SNPs (rs4420638) and then (c) rs769449 as well as (d) rs5167.

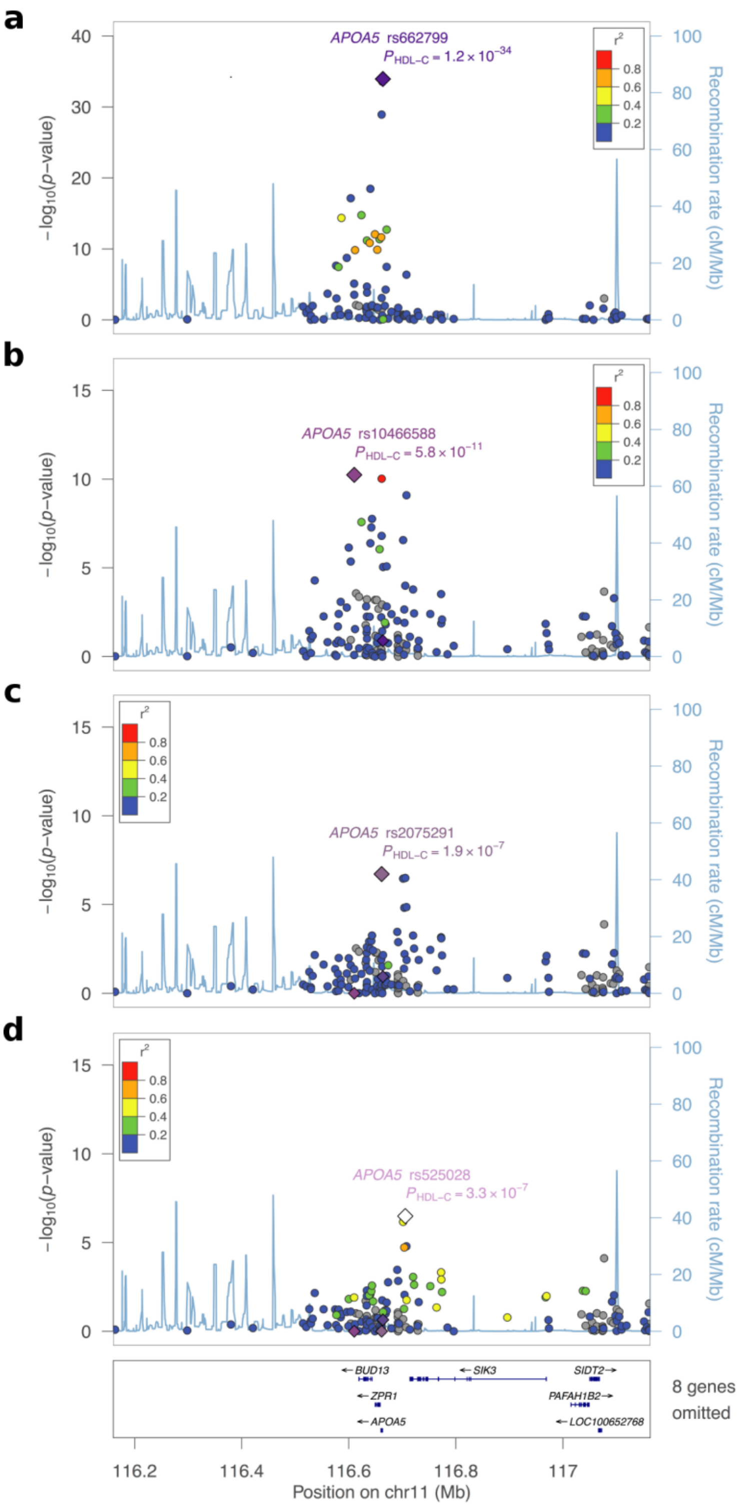

Supplementary Figure 8. Regional plots of conditional analysis between *APOA5* and HDL cholesterol. Association *p*-values are shown (a) before and after stepwise conditioning on (b) known SNPs (rs964184 and rs651821) and (c) rs10466588 as well as (d) rs2075291.

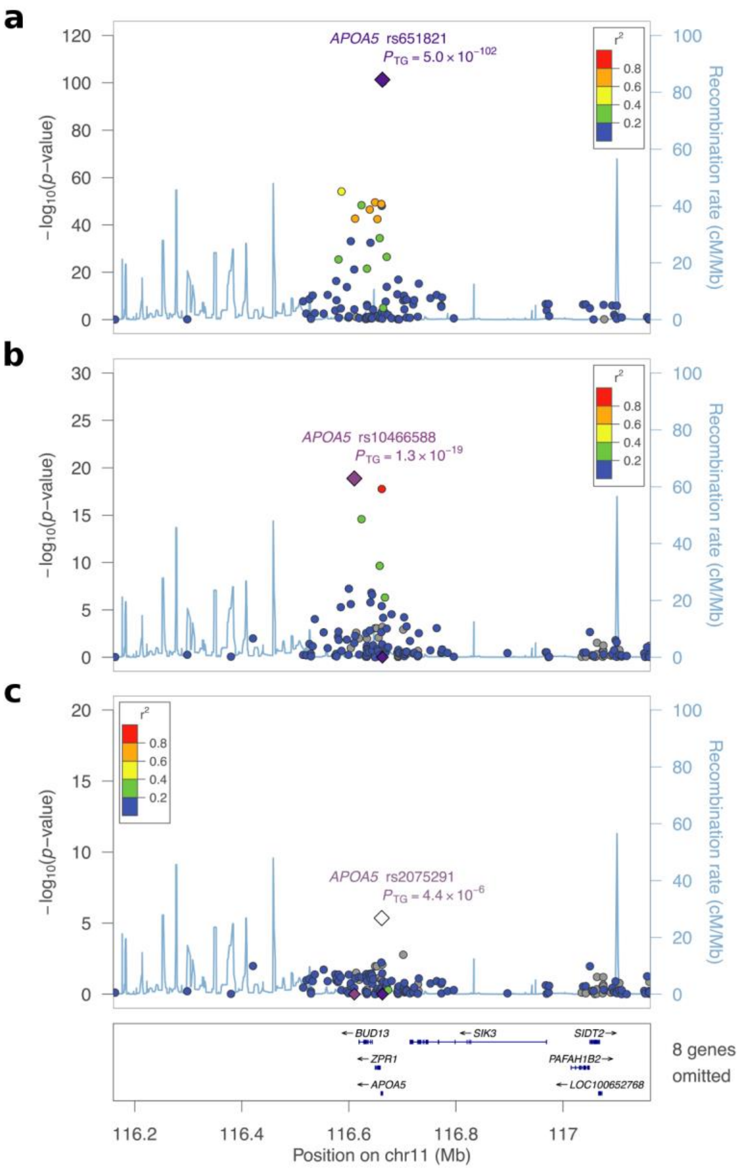

Supplementary Figure 9. Regional plots of conditional analysis between *APOA5* and triglycerides. Association *p*-values are shown (a) before and after stepwise conditioning on (b) known SNPs (rs964184 and rs651821) and then (c) rs10466588.

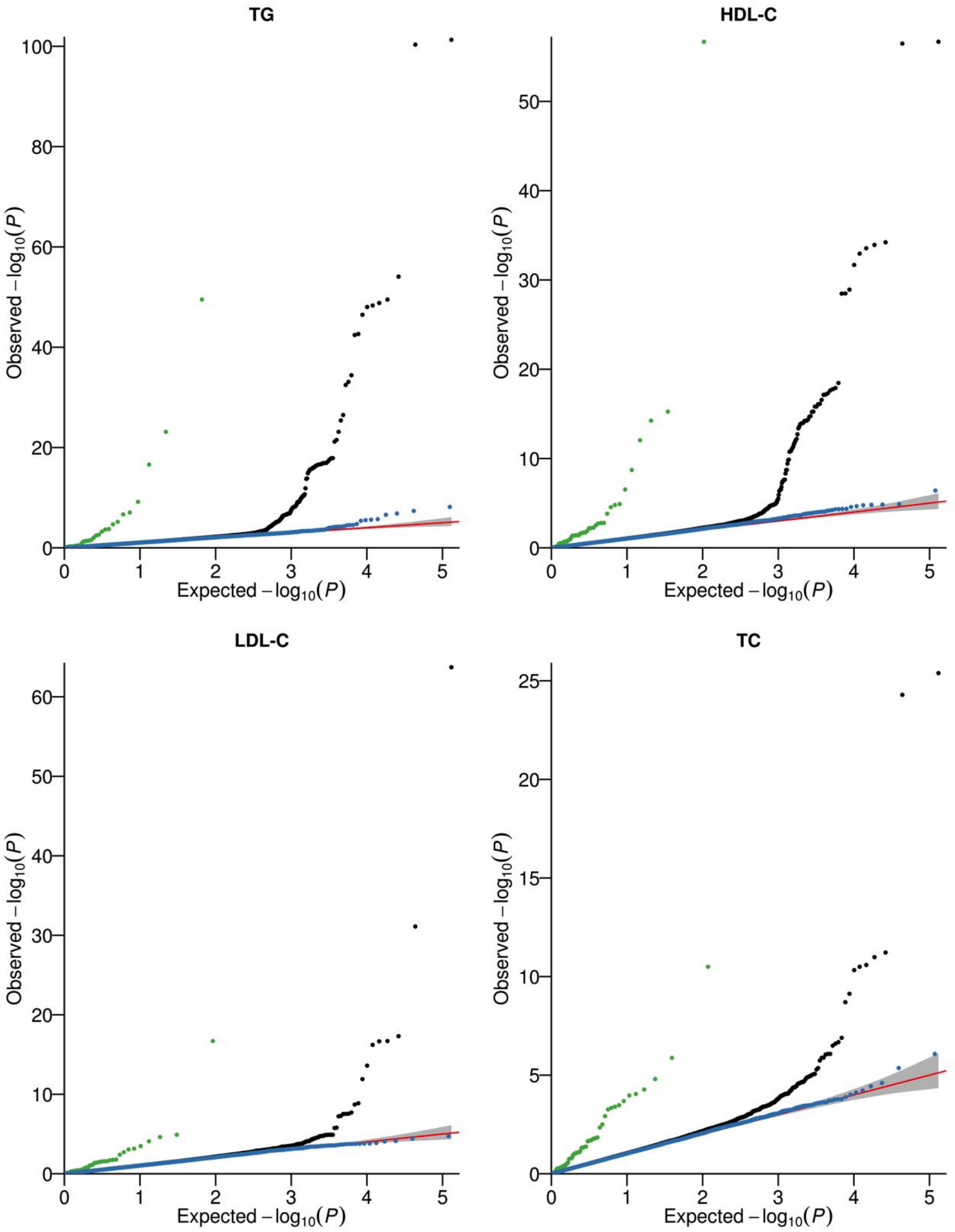

Supplementary Figure 10. Quantile-quantile plots of association P-values for TG, HDL-C, LDL-C and TC. Associations for all SNPs tested are represented in black. Green circles represent associations for 157 loci reported in GLGC *et al.* (2013)[1] while blue circles denotes the association after removal of SNPs mapping to the 157 known loci (±1Mb).

Supplementary Tables

Supplementary Table 1. Summary of polymorphic variants in the combined dataset.

Variant type	Frequency	Number of variants genotyped	Number of custom markers [a]	Number of variants in 1000G	Number of Asian-specific variants [b]
Damaging protein-altering	> 5%	5,160	43 (0.83)	5,121	243 (4.75)
	1-5%	3,964	216 (5.45)	3,862	2,184 (56.55)
	20 copies - 1%	16,812	7,641 (45.45)	11,031	8,351 (75.70)
	1-19 copies	45,440	9,825 (21.62)	21,743	4,715 (21.69)
Non-damaging protein-altering	> 5%	5,929	71 (1.20)	5,886	123 (2.09)
	1-5%	2,347	133 (5.67)	2,300	936 (40.70)
	20 copies - 1%	6,951	2,876 (41.38)	4,849	3,130 (64.55)
	1-19 copies	16,706	3,247 (19.44)	9,222	1,544 (16.74)
Total	> 5%	41,693	17,031 (40.85)	41,548	761 (1.83)
	1-5%	10,757	3,648 (33.91)	10,588	5,018 (47.39)
	20 copies - 1%	26,846	12,524 (46.65)	18,692	13,053 (69.83)
	1-19 copies	65,980	14,400 (21.82)	33,628	7,206 (21.43)
	**Total**	**145,276**	**47,603 (32.77)**	**104,456**	**26,038 (24.93)**

[a] Proportion of custom makers relative to genotyped are shown in bracket
[b] Proportion of Asian-specific makers relative to all present in 1000G are shown in bracket

Supplementary Table 2. Summary of variants included in single-variant association analysis.

Variant type	Frequency	Number of variants genotyped	Number of custom markers [a]	Number of variants in 1000G	Number of Asian-specific variants [b]
Damaging protein-altering	> 5%	4,697	34 (0.72)	4,674	224 (4.79)
	1-5%	3,640	181 (4.97)	3,567	2,014 (56.46)
	20 copies - 1%	15,773	7,163 (45.41)	10,360	7,856 (75.83)
Non-damaging protein-altering	> 5%	5,256	61 (1.16)	5,226	105 (2.01)
	1-5%	2,112	112 (5.3)	2,071	863 (41.67)
	20 copies - 1%	6,466	2,684 (41.51)	4,509	2,918 (64.72)
Total	> 5%	31,100	15,616 (50.21)	31,003	682 (2.2)
	1-5%	9,531	3,257 (34.17)	9,404	4,604 (48.96)
	20 copies - 1%	25,040	11,703 (46.74)	17,413	12,250 (70.35)
	**Total**	**65,671**	**30,576 (46.56)**	**57,820**	**17,536 (30.33)**

[a] Proportion of custom makers relative to genotyped are shown in bracket

[b] Proportion of Asian-specific makers relative to all present in 1000G are shown in bracket

Supplementary Table 3. Summary of association with GWAS SNPs in combined data (N=12,685).

Lipid trait	GWAS variants [a]			SNPs with P<5x10^{-8}			SNPs with P<0.05			SNPs with concordant direction of effect		
	Total	QC+	MAC ≥20 [b]	N (% [c]), Observed	N, Expected	P [d]	N (%), Observed	N, Expected	P	N (%), Observed	N, Expected	P
HDL-C	60	44	43	3 (7.0)	0	1.5x10^{-18}	22 (51.2)	2.15	9.0x10^{-18}	37 (86.0)	21.5	8.2x10^{-7}
LDL-C	30	26	24	1 (4.2)	0	1.2x10^{-6}	9 (37.5)	1.2	1.3x10^{-6}	21 (87.5)	12	1.3x10^{-4}
TC	39	28	28	0 (0)	0	1	8 (28.6)	1.4	4.9x10^{-5}	19 (67.9)	14	4.4x10^{-2}
TG	28	25	24	4 (16.7)	0	6.6x10^{-26}	13 (52.2)	1.2	1.8x10^{-11}	22 (91.7)	12	1.8x10^{-5}
Total	157	123	119	8 (6.7)	0	3.1x10^{-47}	52 (42.7)	5.95	1.5x10^{-35}	99 (83.2)	59.5	4.6x10^{-14}

[a] GWAS variants refer to the index SNPs of the 157 loci associated with primiary lipid traits in GLGC (2013)

[b] SNPs polymorphic in both Chinese cohorts and with minor allele count (MAC) greater than or equal to 20

[c] Number and proportion of SNPs attaining the corresponding significant levels among GWAS variants passing quality controls and with MAC≥20

[d] P values were estimated using binominal distribution to test for excess of variants reaching significant levels or matching the direction with the null hypothesis of no association.

Supplementary Table 4. Summary of association for GWAS SNPs in HKUTRS (N=5,233) and PUUMA-MI (N=5,643).

Lipid trait (Primary)	Study	GWAS variants [a] Total	GWAS variants [a] MAC ≥6 [b]	SNPs with P<5x10^{-8} Number	SNPs with P<5x10^{-8} Percentage(%)	SNPs with P<0.05 Number	SNPs with P<0.05 Percentage(%)
HDL-C	HKUTRS	60	43	1	2.3	11	25.6
	HUNT-MI	60	44	2	4.5	19	43.2
LDL-C	HKUTRS	30	23	1	4.2	5	20.8
	HUNT-MI	30	23	2	7.7	9	34.6
TC	HKUTRS	39	25	0	0	5	17.9
	HUNT-MI	39	29	0	0	8	27.6
TG	HKUTRS	28	23	2	8.3	11	45.8
	HUNT-MI	28	23	3	13.0	9	39.1
Total	HKUTRS	157	119	4	3.4	32	26.9
	HUNT-MI	157	122	7	5.7	45	36.9

[a] GWAS variants refer to the index SNPs of the 157 loci reported in GLGC (2013)

[b] SNPs passing quality control and with minor allele count (MAC) greater than or equal to 6 as evaluated in Holmen *et al.* (2013)

Supplementary Table 5. Association result of known triglycerides associated variants.

Gene	rs ID	Position	REF [a]	ALT	FREQ [b]	Direction [c]	Effect	s.e.	*P*
ANGPTL3	**rs2131925**	**1:63025942**	**G**	**T**	**0.759**	**++**	**0.091**	**0.015**	**6.82×10^{-10}**
GCKR	**rs1260326**	**2:27730940**	**T**	**C**	**0.491**	**--**	**-0.127**	**0.013**	**7.31×10^{-24}**
MSL2L1	rs645040	3:135926622	G	T	0.879	+-	0.030	0.019	0.125
LRPAP1	rs6831256	4:3473139	A	G	0.362	+-	-0.006	0.013	0.666
KLHL8	rs442177	4:88030261	G	T	0.577	++	0.033	0.013	9.36×10^{-3}
MAP3K1	rs9686661	5:55861786	C	T	0.119	-+	0.017	0.020	0.399
VEGFA	rs998584	6:43757896	C	A	0.572	++	0.025	0.013	0.047
TYW1B	rs13238203	7:72129667				not on the exome chip			
MLXIPL	rs17145738	7:72982874	C	T	0.111	--	-0.109	0.020	8.93×10^{-8}
MET	rs38855	7:116358044	A	G	0.584	-+	-0.013	0.013	0.326
PINX1	rs11776767	8:10683929	G	C	0.201	++	0.009	0.016	0.554
NAT2	rs1495741	8:18272881	G	A	0.457	--	-0.044	0.013	5.81×10^{-4}
LPL	**rs12678919**	**8:19844222**	**A**	**G**	**0.091**	**--**	**-0.186**	**0.022**	**2.49×10^{-17}**
TRIB1	rs2954029	8:126490972	A	T	0.567	--	-0.054	0.013	2.08×10^{-5}
AKR1C4	rs1832007	10:5254847	A	G	0.101	-+	-0.020	0.021	0.352
JMJD1C	rs10761731	10:65027610	A	T	0.317	--	-0.010	0.014	0.475
CYP26A1	rs2068888	10:94839642	G	A	0.811	--	-0.061	0.016	1.81×10^{-4}
FADS1-2-3	rs174546	11:61569830	C	T	0.469	++	0.061	0.014	6.62×10^{-6}
APOA1	**rs964184**	**11:116648917**	**G**	**C**	**0.784**	**--**	**-0.229**	**0.015**	**3.11×10^{-50}**
LRP1	rs11613352	12:57792580	C	T	0.094	QC- in PUUMA-MI			
CAPN3	rs2412710	15:42683787	G	A	0.000	Monomorphic in HKUTRS			
FRMD5	rs2929282	15:44245931	A	T	0.053	+-	-0.027	0.028	0.332
PDXDC1	rs3198697	16:15129940	C	T	0.004	-+	-0.019	0.098	0.843
CTF1	rs11649653	16:30918487	C	G	0.902	QC- in PUUMA-MI			

Variants with GWAS significant association are shown in bold

[a] REF and ALT: reference and alternative effector alleles with reference to human refence genome build hg19

[b] FREQ: Alternative effector allele frequencies

[c] Direction refers to the direction of effect estimates of HKUTRS and PUUMA-MI respectively

Supplementary Table 5 (continued). Association result of known triglycerides associated variants.

Gene	rs ID	Position	REF [a]	ALT	FREQ [b]	Direction [c]	Effect	s.e.	*P*
MPP3	rs8077889	17:41878166	A	C	0.003	++	0.231	0.114	0.042
INSR	rs7248104	19:7224431	G	A	0.311	+-	-0.003	0.014	0.800
PEPD	rs731839	19:33899065	G	A	0.463	--	-0.027	0.013	0.035
PLA2G6	rs5756931	22:38546033	T	C	0.121	-+	-0.008	0.019	0.685

Supplementary Table 6. Association result of known HDL cholesterol associated variants.

Gene	rs ID	Position	REF [a]	ALT	FREQ [b]	Direction [c]	Effect	s.e.	*P*
PIGV-NR0B2	rs12748152	1:27138393	C	T	0.024	--	-0.086	0.041	0.038
PABPC4	rs4660293	1:40028180	A	G	0.126	--	-0.052	0.019	$5.68X10^{-3}$
HDGF-PMVK	rs12145743	1:156700651	T	G	0.101	QC- in PUUMA-MI			
ANGPTL1	rs4650994	1:178515312	G	A	0.535	--	-0.016	0.013	0.192
ZNF648	rs1689800	1:182168885	A	G	0.294	--	-0.043	0.014	$1.89X10^{-3}$
GALNT2	rs4846914	1:230295691	G	A	0.213	++	0.036	0.015	0.019
COBLL1	rs12328675	2:165540800	T	C	0.003	--	-0.018	0.124	0.884
CPS1	rs1047891	2:211540507				not on the exome chip			
IRS1	rs2972146	2:227100698	G	T	0.931	--	-0.079	0.025	$1.69x10^{-3}$
ATG7	rs2606736	3:11400249	C	T	0.662	++	0.007	0.013	0.601
SETD2	rs2290547	3:47061183	G	A	0.239	-+	-0.006	0.015	0.703
RBM5	rs2013208	3:50129399	C	T	0.863	QC- in PUUMA-MI			
STAB1	rs13326165	3:52532118	A	G	0.978	++	0.006	0.043	0.895
GSK3B	rs6805251	3:119560606				not on the exome chip			
C4orf52	rs10019888	4:26062990				not on the exome chip			
FAM13A	rs3822072	4:89741269				not on the exome chip			
ADH5	rs2602836	4:100014805	A	G	0.833	--	-0.004	0.017	0.836
SLC39A8	rs13107325	4:103188709	C	T	0.000	Monomorphic in HKUTRS			
ARL15	rs6450176	5:53298025	G	A	0.437	++	0.013	0.013	0.310
RSPO3	rs1936800	6:127436064	C	T	0.520	--	-0.002	0.013	0.881
CITED2	rs605066	6:139829666	C	T	0.298	++	0.022	0.014	0.118
DAGLB	rs702485	7:6449272				not on the exome chip			
SNX13	rs4142995	7:17919258				not on the exome chip			
IKZF1	rs4917014	7:50305863	T	G	0.300	++	0.025	0.014	0.076
KLF14	rs4731702	7:130433384	C	T	0.291	++	0.058	0.014	$2.86x10^{-5}$
TMEM176A	rs17173637	7:150529449	T	C	0.012	+-	-0.070	0.057	0.222
PPP1R3B	rs9987289	8:9183358	A	G	0.988	-+	0.114	0.057	0.045
TRPS1	rs2293889	8:116599199	T	G	0.861	++	0.037	0.018	0.043

Supplementary Table 6 (continued). Association result of known HDL cholesterol associated variants.

Gene	rs ID	Position	REF [a]	ALT	FREQ [b]	Direction [c]	Effect	s.e.	*P*
TTC39B	rs581080	9:15305378	G	C	0.919	+-	0.025	0.023	0.291
ABCA1	**rs1883025**	**9:107664301**	**C**	**T**	**0.224**	**--**	**-0.090**	**0.015**	**$1.93x10^{-9}$**
MARCH8-ALOX5	rs970548	10:46013277	A	C	0.066	++	0.075	0.025	$3.08x10^{-3}$
AMPD3	rs2923084	11:10388782	A	G	0.514	--	-0.016	0.013	0.196
LRP4	rs3136441	11:46743247	T	C	0.577	--	-0.003	0.013	0.789
OR4C46	rs11246602	11:51512090					not on the exome chip		
KAT5	rs12801636	11:65391317	G	A	0.425	++	0.018	0.013	0.159
MOGAT2-DGAT2	rs499974	11:75455021	C	A	0.265		QC- in PUUMA-MI		
PDE3A	rs7134375	12:20473758	C	A	0.270	++	0.029	0.014	0.041
MVK	rs7134594	12:110000193	C	T	0.302	++	0.029	0.014	0.039
SBNO1	rs4759375	12:123796238	C	T	0.285		QC- in PUUMA-MI		
ZNF664	rs4765127	12:124460167	G	T	0.079		QC- in PUUMA-MI		
SCARB1	rs838880	12:125261593	C	T	0.489	--	-0.040	0.013	$1.62x10^{-3}$
ZBTB42-AKT1	rs4983559	14:105277209	G	A	0.137	--	-0.008	0.018	0.662
LIPC	**rs1532085**	**15:58683366**	**A**	**G**	**0.544**	**--**	**-0.104**	**0.013**	**$5.61x10^{-16}$**
LACTB	rs2652834	15:63396867	A	G	0.985	--	-0.048	0.052	0.363
FTO	rs1121980	16:53809247	G	A	0.182	--	-0.038	0.016	0.019
CETP	**rs3764261**	**16:56993324**	**C**	**A**	**0.167**	**++**	**0.272**	**0.017**	**$2.09x10^{-57}$**
LCAT	rs16942887	16:67928042	G	A	0.029	-+	0.037	0.037	0.325
CMIP	rs2925979	16:81534790	T	C	0.588	++	0.049	0.013	$1.51x10^{-4}$
STARD3	rs11869286	17:37813856	G	C	0.418	++	0.035	0.013	$6.13x10^{-3}$
ABCA8	rs4148008	17:66875294	C	G	0.551		QC- in PUUMA-MI		
PGS1	rs4129767	17:76403984	G	A	0.345	++	0.014	0.013	0.285
LIPG	rs7241918	18:47160953	G	T	0.883	++	0.030	0.020	0.129
MC4R	rs12967135	18:57849023	G	A	0.169		QC- in PUUMA-MI		
ANGPTL4	rs7255436	19:8433196	C	A	0.090	++	0.051	0.022	0.022
ANGPTL8	rs737337	19:11347493	T	C	0.274	--	-0.073	0.014	$2.95x10^{-7}$
HAS1	rs17695224	19:52324216	G	A	0.201	--	-0.035	0.016	0.025

Supplementary Table 6 (continued). Association result of known HDL cholesterol associated variants.

Gene	rs ID	Position	REF [a]	ALT	FREQ [b]	Direction [c]	Effect	s.e.	*P*
LILRA3	rs386000	19:54792761	G	C	0.571	QC- in PUUMA-MI			
HNF4A	rs1800961	20:43042364	C	T	0.015	--	-0.108	0.052	0.039
PLTP	rs6065906	20:44554015	T	C	0.025	QC- in PUUMA-MI			
UBE2L3	rs181362	22:21932068	C	T	0.487	+-	-0.016	0.013	0.202

Variants with GWAS significant association are shown in bold
[a] REF and ALT: reference and alternative effector alleles with reference to human refence genome build hg19
[b] FREQ: Alternative effector allele frequencies
[c] Direction refers to the direction of effect estimates of HKUTRS and PUUMA-MI respectively

Supplementary Table 7. Association result of known LDL cholesterol associated variants.

Gene	rs ID	Position	REF [a]	ALT	FREQ [b]	Direction [c]	Effect	s.e.	*P*
PCSK9	rs2479409	1:55504650	G	A	0.311	-+	-0.005	0.014	0.725
SORT1	**rs629301**	**1:109818306**	**G**	**T**	**0.937**	**++**	**0.220**	**0.026**	**$2.01x10^{-17}$**
ANXA9-CERS2	rs267733	1:150958836	A	G	0.035	+-	-0.057	0.035	0.099
APOB	rs1367117	2:21263900	G	A	0.134	++	0.054	0.019	$3.96x10^{-3}$
ABCG5/8	rs4299376	2:44072576	G	T	0.994	--	-0.116	0.083	0.164
EHBP1	rs2710642	2:63149557	G	A	0.696	++	0.058	0.014	$2.41x10^{-5}$
INSIG2	rs10490626	2:118835841	G	A	0.001	++	0.156	0.209	0.455
LOC84931	rs2030746	2:121309488	C	T	0.479	-+	0.009	0.013	0.491
FN1	rs1250229	2:216304384	T	C	0.915	++	0.050	0.023	0.028
CMTM6	rs7640978	3:32533010	C	T	0.055	--	-0.064	0.028	0.020
ACAD11	rs17404153	3:132163200	G	T	0.124	--	-0.018	0.019	0.346
CSNK1G3	rs4530754	5:122855416	G	A	0.347	++	0.027	0.013	0.045
MYLIP	rs3757354	6:16127407	C	T	0.392	--	-0.014	0.013	0.287
HFE	rs1800562	6:26093141	G	A	0.001	Monomorphic in HKUTRS			
LPA	rs1564348	6:160578860	T	C	0.004	-+	-0.009	0.098	0.927
MIR148A	rs4722551	7:25991826	T	C	0.017	QC- in PUUMA-MI			
SOX17	rs10102164	8:55421614	G	A	0.214	++	0.034	0.015	0.027
PLEC1	rs11136341	8:145043543	A	G	0.119	QC- in PUUMA-MI			
ABO	rs9411489	9:136155000	C	T	0.200	++	0.049	0.016	$1.90x10^{-3}$
ST3GAL4	rs11220462	11:126243952	G	A	0.346	++	0.014	0.013	0.289
BRCA2	rs4942486	13:32953388	T	C	0.545	--	-0.018	0.013	0.159
NYNRIN	rs8017377	14:24883887	G	A	0.054	--	-0.006	0.028	0.839
OSBPL7	rs7206971	17:45425115	G	A	0.264	++	0.012	0.014	0.399
APOH-PRXCA	rs1801689	17:64210580	A	C	0.000	Monomorphic in HKUTRS			
LDLR	rs6511720	19:11202306	G	T	0.009	--	-0.163	0.068	0.016
APOE	rs4420638	19:45422946	A	G	0.102	QC- in PUUMA-MI			
SPTLC3	rs364585	20:12962718	A	G	0.577	++	0.010	0.013	0.441
SNX5	rs2328223	20:17845921				not on the exome chip			

Supplementary Table 7 (continued). Association result of known LDL cholesterol associated variants.

Gene	rs ID	Position	REF [a]	ALT	FREQ [b]	Direction [c]	Effect	s.e.	*P*
TOP1	rs6029526	20:39672618	T	A	0.820	++	0.017	0.016	0.313
MTMR3	rs5763662	22:30378703	C	T	0.104	++	0.037	0.021	0.075

Variants with GWAS significant association are shown in bold

[a] REF and ALT: reference and alternative effector alleles with reference to human refence genome build hg19

[b] FREQ: Alternative effector allele frequencies

[c] Direction refers to the direction of effect estimates of HKUTRS and PUUMA-MI respectively

Supplementary Table 8. Association result of known total cholesterol associated variants.

Gene	rs ID	Position	REF [a]	ALT	FREQ [b]	Direction [c]	Effect	s.e.	*P*
ASAP3	rs1077514	1:23766233	C	T	0.668	+-	-0.009	0.013	0.516
LDLRAP1	rs12027135	1:25775733	A	T	0.295	++	0.024	0.014	0.082
EVI5	rs7515577	1:93009438	C	A	0.960	-+	0.021	0.032	0.516
MOSC1	rs2642442	1:220973563				not on the exome chip			
IRF2BP2	rs514230	1:234858597	A	T	0.212	QC- in PUUMA-MI			
RAB3GAP1	rs7570971	2:135837906	C	A	1.000	QC- in PUUMA-MI			
ABCB11	rs2287623	2:169830155	G	A	0.755	+-	-0.020	0.015	0.167
FAM117B	rs11694172	2:203532304	A	G	0.148	++	0.017	0.018	0.330
UGT1A1	rs11563251	2:234679384	C	T	0.093	--	-0.031	0.022	0.153
RAF1	rs2290159	3:12628920				not on the exome chip			
PXK	rs13315871	3:58381287	G	A	0.002	+-	0.020	0.139	0.884
HMGCR	rs12916	5:74656539	T	C	0.530	QC- in PUUMA-MI			
TIMD4	rs6882076	5:156390297	T	C	0.731	++	0.058	0.014	$5.34x10^{-5}$
HLA	rs3177928	6:32412435	G	A	0.047	++	0.023	0.030	0.440
C6orf106	rs2814982	6:34546560	C	T	0.080	++	0.039	0.023	0.089
KCNK17	rs2758886	6:39250837				not on the exome chip			
FRK	rs9488822	6:116312893				not on the exome chip			
HBS1L	rs9376090	6:135411228	T	C	0.283	+-	-0.015	0.014	0.271
GPR146	rs1997243	7:1083777	A	G	0.002	-+	0.116	0.137	0.397
DNAH11	rs12670798	7:21607352	T	C	0.490	-+	0.000	0.013	0.997
NPC1L1	rs2072183	7:44579180	G	C	0.367	-+	0.002	0.013	0.848
CYP7A1	rs2081687	8:59388565	T	C	0.793	--	-0.060	0.015	$1.08x10^{-4}$
VLDLR	rs3780181	9:2640759	A	G	0.093	--	-0.039	0.022	0.072
VIM-CUBN	rs10904908	10:17260290	A	G	0.681	-+	0.027	0.013	0.048
GPAM	rs2255141	10:113933886	A	G	0.700	--	-0.034	0.014	0.014
SPTY2D1	rs10128711	11:18632984	T	C	0.519	QC- in PUUMA-MI			
PHLDB1	rs11603023	11:118486067	T	C	0.744	-+	0.004	0.014	0.768
UBASH3B	rs7941030	11:122522375	T	C	0.284	++	0.012	0.014	0.380

Supplementary Table 8 (continued). Association result of known total cholesterol associated variants.

Gene	rs ID	Position	REF [a]	ALT	FREQ [b]	Direction [c]	Effect	s.e.	*P*
PHC1-A2ML1	rs4883201	12:9082581	A	G	0.324	--	-0.023	0.013	0.083
BRAP	rs11065987	12:112072424	A	G	0.003	--	-0.269	0.112	0.017
HNF1A	rs1169288	12:121416650	A	C	0.413	++	0.047	0.013	2.06×10^{-4}
HPR	rs2000999	16:72108093	G	A	0.258	QC- in PUUMA-MI			
DLG4	rs314253	17:7091650	T	C	0.482	+-	-0.021	0.013	0.091
CILP2	rs10401969	19:19407718	T	C	0.093	--	-0.076	0.022	4.57×10^{-4}
FLJ36070	rs492602	19:49206417	A	G	0.009	+-	-0.004	0.068	0.957
ERGIC3	rs2277862	20:34152782				not on the exome chip			
MAFB	rs2902940	20:39091487	A	G	0.327	QC- in PUUMA-MI			
TOM1	rs138777	22:35711098	A	G	0.535	--	-0.037	0.013	3.16×10^{-3}
PPARA	rs4253772	22:46627603	C	T	0.001	--	-0.223	0.167	0.181

[a] REF and ALT: reference and alternative effector alleles with reference to human refence genome build hg19
[b] FREQ: Alternative effector allele frequencies
[c] Direction refers to the direction of effect estimates of HKUTRS and PUUMA-MI respectively

Supplementary Table 9. Clinical characteristics of subjects involved in quantitative blood lipids analyses.

	Subjects involved in blood lipids analyses
Number	5233
Male (%)	57.80
Age* (year)	58.9 ± 11.9
BMI (kg/m2)	25.39 ± 4.18
CAD (%)	36.70
T2DM (%)	65.60
HT (%)	68.50
SBP† (mmHg)	140.37 ± 23.30
DBP† (mmHg)	77.64 ± 10.63
Dyslipidemia (%)	65.60
Use of lipid lowering drug (%)	51.00
TC (mmol/L)	5.16 ± 1.10
HDL-C‡(mmol/L)	1.19(0.98-1.45)
LDL-C (mmol/L)	3.15 ± 0.95
TG‡ (mmol/L)	1.32(0.91-2.00)
Current Smoker (%)	11.5

Data as mean ± standard deviation or median with interquartile range.
*Age is defined as the age at blood lipid tested.
†SBP added 10mmHg and DBP added 5mmHg if on anti-hypertensive drug.
‡Natural-log-transformed before analysis.
CAD: Coronary artery disease; T2DM: Type 2 diabetes mellitus; BMI: Body mass index; TC: Total cholesterol; HDL-C: High-density lipoprotein cholesterol; LDL-C: Low-density lipoprotein cholesterol; TG: Triglyceride; HT: Hypertension. Pre- or without treatment lipid levels are shown. Subjects with the use of lipid-lowering drug but with no record of pre-treatment lipid levels were excluded from the quantitative blood lipids study. Dyslipidaemia is defined as having one or more of the following criteria: (1) TG ≥ 1.7mmol/L; (2) HDL-C < 1.04 in males and < 1.29mmol/L in females; (3) LDL ≥ 3.4mmol/L; and (4) already on lipid-lowering drugs.

Supplementary Table 10. Clinical characteristics of subjects involved in CAD case-control analysis.

	Non-CAD	CAD	*P*-values
Number	3388	2372	-
Male (%)	48.60	74.10	<0.001
Age* (year)	58.34 ± 12.25	62.17 ± 10.62	<0.001
BMI (kg/m2)	25.30 ± 4.43	25.60 ± 3.66	0.010
T2DM (%)	63.00	68.80	<0.001
HT (%)	60.30	82.20	<0.001
SBP† (mmHg)	137.22 ± 23.36	145.13 ± 21.75	<0.001
DBP† (mmHg)	77.92 ± 10.53	76.89 ± 11.04	0.001
Dyslipidemia (%)	53.90	89.40	<0.001
Use of lipid lowering drug (%)	27.20	95.70	<0.001
TC (mmol/L)	5.13 ± 1.05	5.23 ± 1.17	0.001
HDL-C‡(mmol/L)	1.25(1.03-1.52)	1.09(0.90-1.30)	<0.001
LDL-C (mmol/L)	3.10 ± 0.90	3.26 ± 1.02	<0.001
TG‡(mmol/L)	1.24(0.88-1.90)	1.50(1.10-2.20)	<0.001
Current Smoker (%)	10.30	13.40	<0.001

Data as mean ± standard deviation or median with interquartile range.
*Age is defined by the age at diagnosis for cases and the age at recruitment for controls.
†SBP added 10mmHg and DBP added 5mmHg if on anti-hypertensive drug.
‡Natural-log-transformed before analysis.
CAD: Coronary artery disease; T2DM: Type 2 diabetes mellitus; BMI: Body mass index; TC: Total cholesterol; HDL-C: High-density lipoprotein cholesterol; LDL-C: Low-density lipoprotein cholesterol; TG: Triglyceride; HT: Hypertension. Pre- or without treatment lipid levels are shown. Subjects with the use of lipid-lowering drug but with no record of pre-treatment lipid levels were excluded from the quantitative blood lipids study. Dyslipidaemia is defined as having one or more of the following criteria: (1) TG ≥ 1.7mmol/L; (2) HDL-C < 1.04 in males and < 1.29mmol/L in females; (3) LDL ≥ 3.4mmol/L; and (4) already on lipid-lowering drugs.

Supplementary Table 11. Cohort descriptions of PUUMA-MI and HUNT-MI.

Trait	Mean value (S.D.)	
	**PUUMA-MI (N=7,452)**	**HUNT-MI (N=5,643)**
Ancestry	Chinese	Norwegian
Age (year)	58.5 (9.6)	79.2 (12.4)
Female	5,556 (55.6%)	2,003 (33.7%)
BMI (kg/m^2)	26.8 (4.4)	26.9 (3.7)
LDL-C (mg/dL)	119.1 (35.2)	160.1 (43.3)
HDL-C (mg/dL)	52.2 (17.0)	51.0 (15.1)
TC (mg/dL)	197.2 (52.2)	246.3 (47.6)
TG (mg/dL)	145.3 (107.2)	178.9 (103.4)
SBP (mg/dL)	133.0 (17.4)	145.2 (21.9)
DBP (mg/dL)	76.2 (19.0)	83.5 (11.7)
MI Cases	1,700 (19.5%)	2,969 (50.0%)

Values are expressed as mean with standard deviation in parenthesis.
MI: Myocardial infarction

Supplementary Note 1

The University of Hong Kong Theme-based Research Scheme (HKU-TRS)

Hong Kong Chinese CAD Cohort. The Hong Kong Chinese coronary artery disease (CAD) cohort is an on-going prospective cohort study on the risk factors and clinical outcomes in Chinese patients with established CAD. This study was first started in 2004-2005 in the Queen Mary Hospital. In brief, consecutive patients underwent invasive coronary angiogram for assessment and treatment of CAD in the Queen Mary Hospital were screened. Those patients suffered from significant CAD with at least 50% stenosis in one or more of the epicardial coronary artery as determined by coronary angiogram were invited to join this study. Detailed anthropometric and demographic data, including major cardiovascular risk factors and medical, drug and family histories were collected during their hospital admission or in the outpatient clinic follow-up. After an overnight fast, blood samples were drawn, with written informed consent, for DNA and biochemical analyses. Ethical approval was obtained from the Institutional Review Board of the University of Hong Kong/Hospital Authority Hong Kong West Cluster.

Hong Kong Cardiovascular Risk Factors Prevalence Study (CRISPS). The Hong Kong Cardiovascular Risk Factors Prevalence Study (CRISPS) is an on-going population-based prospective study of cardiovascular risk factors in Hong Kong[2]. Details of the CRISPS cohort were previously described[3-6]. In brief, CRISPS was first commenced as a population-based survey in 1995-1996 (CRISPS1). 2895 unrelated subjects of Chinese ancestry were selected randomly by their telephone numbers from the Hong Kong population, and were invited to undergo a comprehensive assessment of cardiovascular risks at the Queen Mary Hospital. After the baseline assessment, subjects were invited to participate in the prospective follow-up assessments for the development of major cardiovascular risk factors in 2000-2004 (CRISPS2), 2005-2008 (CRISPS3) and 2010-2012 (CRISPS4). At each follow-up visit, subjects' anthropometric and demographic data were collected. A detailed questionnaire was used to record the subjects' medical, drug and family histories of major cardiovascular risk factors. Fasting venous blood were obtained after an overnight fast for the measurement of lipids and glucose levels. A 75-gram OGTT was performed in all subjects not on anti-diabetic medications. Buffy coat were collected for DNA extraction. Serum and plasma samples were stored in aliquots at -80℃ for biochemical analyses. All participants gave written informed consent and the study protocol was approved by the Institutional Review Board of the University of Hong Kong/Hospital Authority Hong Kong West Cluster.

Hong Kong West Diabetes Registry (HKWDR). The Hong Kong West Diabetes Registry (HKWDR) was commenced in August 2008. All patients with type 2 diabetes who were on regular follow-up at the medical specialist clinics of the Hong Kong West Cluster were invited to undergo comprehensive clinical assessments and laboratory investigations to determine their control of diabetes and related cardiovascular risk factors, and the presence of diabetic complications, including cardiovascular diseases, diabetic retinopathy, and diabetic nephropathy. The subjects' anthropometric and demographic data were collected. A detailed questionnaire was used to record the subjects' medical, drug and family histories of major cardiovascular risk factors. After an overnight fast, blood samples were drawn, with written informed consent, for DNA and biochemical analyses. Ethical approval was obtained from the Institutional Review Board of the University of Hong Kong/Hospital Authority Hong Kong West Cluster.

The Joint Institute of the Peking University Health Science Center and the University of Michigan Medical School study of Myocardial Infarction (PUUMA-MI)

PUUMA-MI is a large-scale project designed to study cardiovascular disease and related traits, including myocardial infarction (MI) and plasma lipid levels, in China. For samples collected from Peking University Third Hospital, blood samples were taken in the morning after an overnight fast and collected into vacuum tubes containing EDTA for the measurement of plasma lipids. Clinical chemical analyses were conducted at the central chemistry lab of Peking University Third Hospital. Using Beckman Coulter AU 5800 Auto-Analyzer (Tokyo, Japan), total cholesterol (TC) was measured by an enzymatic method (Baiding Biological Engineering Ltd, Beijing, China); triglycerides (TG) were measured by an enzymatic (with peroxidase) method (Biosino Bio-Technology Co. Ltd, Beijing, China); and high-density lipoprotein cholesterol (HDL-C) and low-density lipoprotein cholesterol (LDL-C) were measured by a liquid selective detergent method (Sekisui Medical Co. Ltd, Tokyo, Japan). The day-to-day coefficients of variation were 0.9%-2.0% for TC, 1.6% for HDL-C, 1.5% for LDL-C and 0.8% - 2.1% for TG. For samples from Peking University First Hospital biorepository, fasting plasma lipid levels (including serum TC, LDL-C, HDL-C and TG) were tested using Roche cobas 8000 modular analyzer series (Indianapolis, IN, USA) in Beijing Shijingshan cohort samples (N=836) and Beckman coulter UniCel DxC 800 Synchron (Brea, CA, USA) in Peking University First Hospital-based samples (N=7,339) after overnight fasting, respectively.

Supplementary Methods

Asian Exomechip

The Illumina Infinium HumanExome BeadChip (HumanExome-12v1_A) was primarily designed for examining 242,901 markers, including >200,000 protein-altering variants (non-synonymous, stop and splicing) identified from ~12,000 sequenced genomes and exomes. It also included >20,000 non-exonic variants contributed by multiple consortia, e.g. NHLBI Exome Sequencing Project, as well as variants designed for sample tracking, ancestry differentiation and establishing segments of identity by descent (IBD) (http://genome.sph.umich.edu/wiki/Exome_Chip_Design). While the majority of the discovery sequencing exomes are of European ancestry, the rare variants included are constrained to those observed mainly in Europeans. To augment the array with variants more relevant to Chinese population, we further included approximately 30K markers identified from three exome sequencing projects on ~1000 Chinese samples. Additionally, a custom set of 29,930 variants for fine-mapping or GWAS follow-up studies were added. The third custom panel of 1,501 SNPs specifically designed for candidate gene association analysis at HKU-TRS was not shared between cohorts, thereby not considered in the meta-analysis.

Data quality controls

HKU-TRS. For individual-based quality control, we first removed 11 samples with low call rate (<98%) and 24 outliers with respect to genotype heterozygosity (inbreeding coefficient deviating >4 standard deviation from the mean). Relatedness check identified 40 duplicated and 178 related samples to be removed. Forty samples have problems of gender mismatch, most of which are kept in the same storage container. As a precautionary measure, we also excluded individuals (n=23) stored alongslide with these samples to avoid mislabeling. In addition, gender check highlighted 13 males with possible Klinefelter syndrome. These samples were further excluded from the subsequent analysis. To investigate the existence of non-Chinese samples, we performed a principal component analysis using a panel of 22,204 independent common SNPs (MAF>0.05), 8 outliers were thus removed. Among the 5,763 samples passing quality control, we further excluded 530 subjects who have received lipid-lowering medication but with no record of pre-treatment lipid level. We further employed a SNP-based quality control that removed SNPs with >2% missingness (n=4,072) or violating Hardy-Weinberg equilibrium (P<1x10^{-5}; n=159). While our analysis aims to identify functional variants affecting plasma lipid levels, we excluded 8,086 non-protein altering SNPs that were originally designed for quality controls. These include ancestry informative loci (AIMs) for distinguishing Europeans from native and African Americans, grid

SNPs for identifying IBD segments and fingerprint SNPs for sample tracking. After all quality control measures,, 5,233 samples and 286,795 SNPs, of which 176,149 variants are monomorphic, remained in the dataset and were subject to association analysis.

PUUMA-MI.To obtain high quality genotypes, strict criteria were applied to filter out low quality genotypes. We performed plate-level, sample-level and variant-level checks to exclude poor quality genotype calls from the dataset. First, we examined the call rates for each plate, and dropped seven plates (672 samples) showing significant lower call rates and higher heterozygosity than other plates. Next, by examining the genotyping quality of each individual, we excluded 73 samples with low call rates (<99%) and 122 samples with mismatched gender between genotype and medical record. We identified and excluded 145 samples with higher than expected levels of contamination by performing BAFRegress[7]. For each of 377 pairs of duplicated samples, the sample with the highest call rate was retained. Furthermore, 1,189 individuals with unknown or ambiguous diagnosis of cardiovascular disease were also excluded from analyses. Last, marker-level quality control was performed to exclude 496 variants with low cluster score (<0.4), 17,245 with low call rate (<99.9%) and 2,021 that deviated from Hardy-Weinberg equilibrium ($P<1x10^{-4}$). Finally, 7,452 samples and 282,456 markers were retained after quality control. Among the successfully genotyped markers, 129,306 are polymorphic in the Chinese samples. To further verify marker allele assignment, we examined the correlation between allele frequency of the PUUMA-MI Chinese samples and those of East Asian samples from 1000 Genomes Project, and a high correlation (r^2=0.98) was observed.

Annotation

Functions of variants as well as protein changes for nonsynonymous SNPs were annotated by KGGseq according to RefGene annotation[8]. In case of various functions denoted by different isoforms, the most damaging change together with the corresponding mRNA accession was represented. Allele frequency information of the Europeans and Africans was first retrieved from the Exome Variant Server (EVS) data release (ESP6500SI-V2; http://evs.gs.washington.edu/EVS/) based on 6503 samples drawn from NHLBI Exome Sequencing Project (ESP) and was then integrated into the annotation of KGGseq.

For non-coding variants, we looked up the possible regulatory functions using Regulome[9] and SNPs overlapping DNaseI hypersensitivity sites or transcriptional-related chromatin signals were further examined in the relevant cell line HepG2 of The Encyclopedia of DNA Elements (ENCODE Mar 2012

Freeze)[10] via UCSC Genome Browser. Expression QTL (eQTL) information was checked against eQTL browser.

Supplementary References

1. Global Lipids Genetics, C. *et al.* Discovery and refinement of loci associated with lipid levels. *Nat Genet* **45**, 1274-83 (2013).
2. Janus, E.D. Epidemiology of cardiovascular risk factors in Hong Kong. *Clin Exp Pharmacol Physiol* **24**, 987-8 (1997).
3. Cheung, B.M. *et al.* Development of diabetes in Chinese with the metabolic syndrome: a 6-year prospective study. *Diabetes Care* **30**, 1430-6 (2007).
4. Chen, C. *et al.* High plasma level of fibroblast growth factor 21 is an Independent predictor of type 2 diabetes: a 5.4-year population-based prospective study in Chinese subjects. *Diabetes Care* **34**, 2113-5 (2011).
5. Chow, W.S. *et al.* Elevated circulating adipocyte-fatty acid binding protein levels predict incident cardiovascular events in a community-based cohort: a 12-year prospective study. *J Am Heart Assoc* **2**, e004176 (2013).
6. Cheung, C.Y. *et al.* Adiponectin gene variants and the risk of coronary heart disease: a 16-year longitudinal study. *Eur J Endocrinol* **171**, 107-15 (2014).
7. Jun, G. *et al.* Detecting and estimating contamination of human DNA samples in sequencing and array-based genotype data. *Am J Hum Genet* **91**, 839-48 (2012).
8. Li, M.X., Gui, H.S., Kwan, J.S., Bao, S.Y. & Sham, P.C. A comprehensive framework for prioritizing variants in exome sequencing studies of Mendelian diseases. *Nucleic Acids Res* **40**, e53 (2012).
9. Joshi-Tope, G. *et al.* Reactome: a knowledgebase of biological pathways. *Nucleic Acids Res* **33**, D428-32 (2005).
10. Consortium, E.P. An integrated encyclopedia of DNA elements in the human genome. *Nature* **489**, 57-74 (2012).
